# Hot Electron‐Assisted Noble‐Metal‐Free Synergistic Photothermal Catalyst for Solar‐Driven Wastewater Remediation and Microbial Disinfection

**DOI:** 10.1002/advs.202515018

**Published:** 2025-12-12

**Authors:** Manish Kumar Sharma, Bishal Kumar Nahak, Parag Parashar, Uday Kumar Singh, Arshad Khan, Jaba Roy Chowdhury, Parthasarathi Pal, Dongwhi Choi, Hae Gyun Lim, Yu‐Lun Chueh, Zong‐Hong Lin

**Affiliations:** ^1^ Department of Biomedical Engineering National Taiwan University Taipei 10617 Taiwan; ^2^ Department of Materials Science and Engineering National Tsing Hua University Hsinchu 30013 Taiwan; ^3^ International Intercollegiate PhD Program National Tsing Hua University Hsinchu 30013 Taiwan; ^4^ Department of Physics National Sun Yat‐Sen University Kaohsiung 80424 Taiwan; ^5^ Department of Materials Science and Engineering Korea University Seoul 02841 Republic of Korea; ^6^ Department of Mechanical Engineering (Integrated Engineering Program) Kyung Hee University Gyeonggi‐do 17104 Republic of Korea; ^7^ Department of Biomedical Engineering and Smart Gym‑Based Translational Research Center for Active Senior Healthcare Pukyong National University Busan 48513 Republic of Korea

**Keywords:** disinfection, photothermal, ROS generation, thermocatalysis, wastewater treatment

## Abstract

The escalating challenge of water contamination by recalcitrant organic pollutants and pathogens calls for sustainable, solar‐powered technologies that operate without external energy or chemical inputs. Such systems require multifunctional materials capable of harvesting broad‐spectrum sunlight to generate reactive oxygen species (ROS) for simultaneous degradation and disinfection. Conventional photocatalysts are hindered by their limited spectral absorption and rapid charge recombination, which restricts their practical efficacy in real‐world applications. To overcome these challenges, we engineered a hybrid Bi_2_Te_3_@CdS nanostructure incorporated into a porous polyurethane (PU) foam scaffold, facilitating synergistic photothermal and thermocatalytic efficacy under comprehensive solar illumination. The hybrid architecture facilitates effective separation of photogenerated charge carriers, markedly diminishing recombination losses and augmenting the production of ROS, such as •O_2_^−^, •OH, and H_2_O_2_. Concurrently, Bi_2_Te_3_ functions as a thermoelectric absorber that effectively transforms NIR‐induced heat into catalytic activation energy, thereby enhancing degradation kinetics. This dual‐mode activation causes organic pollutants (such as dyes and pesticides) to mineralize quickly and inactivate *E. coli* and *S. aureus* with >99% photothermal assistance. High photostability and reusability enable the material to maintain its activity over multiple cycles without appreciable degradation. By synergistically integrating broadband solar harvesting, efficient ROS generation, and thermocatalytic activation, this study presents an energy‐autonomous strategy for water remediation and sustained antimicrobial defense, offering significant potential for public health benefits.

## Graphical abstract

A multifunctional Bi_2_Te_3_@CdS hybrid system is presented for solar‐driven wastewater remediation and indoor disinfection. The catalyst coated on PU foam efficiently harvests visible‐NIR light to generate heat and reactive oxygen species, enabling the rapid mineralization of organic pollutants. Simultaneously, when applied to glass, the photothermal effect induces temperature elevation and continuous ROS generation, achieving effective antibacterial action. This dual‐function platform provides an energy‐autonomous, recyclable strategy for environmental cleanup and pathogen control.

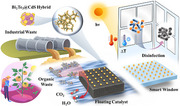

## 1 Introduction

Rapid industrialization and intensive agriculture have led to widespread contamination of water by organic dyes, pesticides, and microorganisms, posing significant environmental and public health concerns.^[^ ^1^, ^2^ ^]^ These contaminants are often chemically resilient and environmentally persistent, making their removal challenging and exacerbating ecological and toxicological risks. Conventional treatment methods such as chlorination, coagulation, and adsorption often result in incomplete removal and may produce secondary toxic byproducts.^[^ ^3^, ^4^, ^5^ ^]^ Solar‐driven advanced oxidation provides a green alternative, where photocatalysts utilize sunlight to mineralize organic pollutants, facilitate water treatment, disinfection, energy generation, and the degradation of emerging contaminants.^[^ ^6^, ^7^, ^8^, ^9^, ^10^, ^11^ ^]^ While photothermal materials convert sunlight to heat for thermal disinfection, enhancing catalytic reactions, H_2_ generation, CO_2_ reduction, etc.^[^ ^12^, ^13^, ^14^, ^15^ ^]^ On the other side, traditional photocatalysts (e.g., metal oxides, metal sulfides, g‐C_3_N_4_, MOFs) are limited by their wide bandgaps, poor light absorption, fast charge recombination, and instability.^[^ ^16^, ^17^, ^18^, ^19^, ^20^, ^21^ ^]^ Noble metal‐based catalysts (e.g., Au, Ag, Pd, Pt) have demonstrated high efficiency in plasmonic photothermal applications due to their pronounced surface plasmon resonance (SPR) effects, which enhance light absorption and localized photothermal conversion.^[^ ^22^, ^23^, ^24^ ^]^ However, their practical implementation is constrained by high material costs and limited natural abundance.^[^ ^25^ ^]^ This underscores the need for earth‐abundant alternatives that maximize solar utilization across the visible and near‐infrared (Vis‐NIR) spectrum.

In this context, bismuth telluride (Bi_2_Te_3)_ emerges as a well‐known topological insulator with a narrow bandgap (≈0.15–0.2 eV).^[^ ^26^, ^27^, ^28^ ^]^ Its high thermal conductivity and strong infrared absorption make it particularly attractive for thermal and photothermal applications, including thermoelectric‐assisted catalysis such as disinfection and wound healing.^[^ ^29^, ^30^, ^31^, ^32^, ^33^, ^34^ ^]^ However, its limited redox potential restricts its standalone use in oxidative thermocatalytic processes. To overcome this limitation and complement the photothermal properties of Bi_2_Te_3_, cadmium sulfide (CdS) serves as a suitable counterpart. CdS is a visible‐light‐active, direct bandgap semiconductor (≈2.4 eV) which is widely explored for photocatalytic applications, particularly in the degradation of organic pollutants and water splitting.^[^ ^35^, ^36^ ^]^ Despite strong light absorption and favorable band positions for redox reactions, CdS suffers from rapid electron‐hole recombination and is prone to photocorrosion under prolonged irradiation, which limits its catalytic stability and reusability.^[^ ^37^, ^38^ ^]^

To address the aforementioned limitations, we employed a heterostructure composed of 2D Bi_2_Te_3_ nanoplates integrated with 3D CdS microflowers. The incorporation of 3D nanoparticles is particularly advantageous due to their inherently high catalytic efficiency, which arises from their increased surface area and enhanced active site availability.^[^ ^39^, ^40^ ^]^ This synergistic 2D/3D architecture is designed to maximize interfacial contact and facilitate more efficient catalytic processes. By integrating Bi_2_Te_3_ with CdS into a heterostructure, efficient charge separation can be achieved, effectively minimizing electron‐hole recombination. Simultaneously, the strong photothermal effect of Bi_2_Te_3_ enhances local temperatures under solar irradiation, thereby accelerating the kinetics of the catalytic reaction. An essential consideration in photo and photothermal catalysis is the selection of an appropriate support scaffold, which significantly impacts light accessibility, catalyst dispersion, mass transfer efficiency, and overall structural stability.^[^ ^41^ ^]^ In this regard, porous polyurethane (PU) foam serves as an excellent substrate for immobilizing catalysts, offering a floating platform with high surface area and easy recoverability.^[^ ^42^, ^43^ ^]^ In our system, oxygen‐plasma treatment of PU foam surface enhances the adhesion of Bi_2_Te_3_@CdS hybrid particles, ensuring uniform coverage and long‐term operational durability. Additionally, the open‐cell architecture of PU foam promotes effective light transmission and mass transport of reactants, thereby improving the accessibility of active sites and enhancing catalytic efficiency. This structural advantage significantly accelerates the degradation of persistent pollutants, including industrial dyes and agricultural pesticides, making it highly suitable for advanced wastewater treatment applications.

Beyond water remediation, solar‐induced ROS generation has been extensively utilized in antimicrobial surface coatings, such as TiO_2_‐based “self‐cleaning” windows, which are known for inactivating microbes under UV light.^[^ ^44^ ^]^ Although advancements have led to visible‐light‐responsive variants with improved antibacterial performance, their effectiveness remains limited to daylight hours.^[^ ^45^, ^46^ ^]^ Bi_2_Te_3_@CdS hybrid system addresses this limitation by enabling continuous disinfection. CdS utilizes visible light during the day for ROS‐mediated antimicrobial activity, while at night, the system leverages ambient environmental energy to sustain oxidative processes. This enables the coated surfaces to maintain effective, sunlight‐independent antibacterial action, making them highly suitable for 24/7 self‐disinfecting applications in healthcare and public environments. In summary, Bi_2_Te_3_@CdS hybrid targets two pressing needs: solar‐driven degradation of organic/waste pollutants and sustainable antimicrobial surface coatings for room disinfection.

## 2 Results and Discussion

### 2.1 Synthesis and Morphological Characterizations of Bi_2_Te_3_@CdS Hybrid

**Figure 1:**
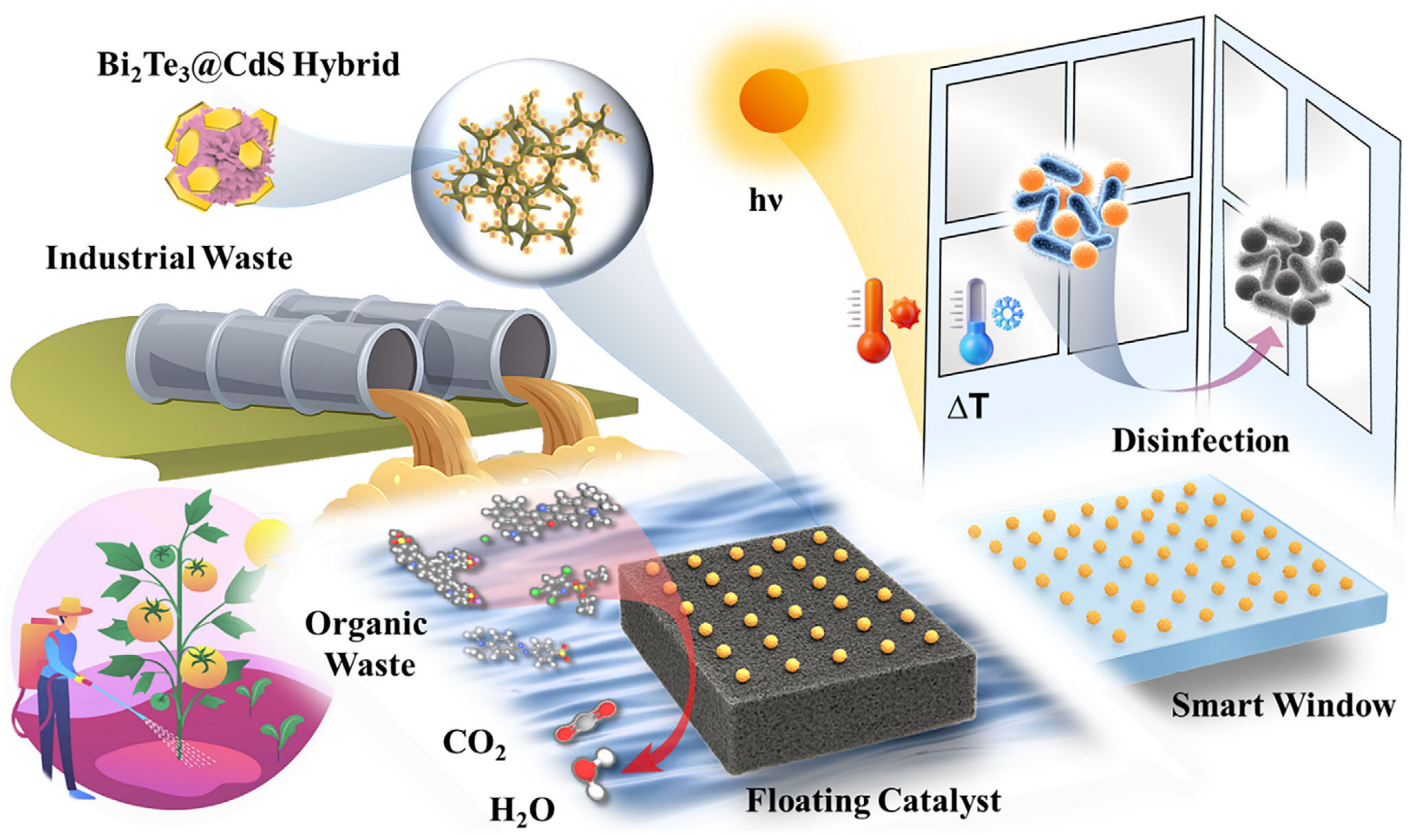
Schematic illustration of Bi_2_Te_3_@CdS hybrid‐based catalysts coated on PU foam (as a floating catalyst) and glass (as a smart window) for wastewater treatment and room disinfection, respectively.

**Figure** **1** illustrates the multifunctional application of the Bi_2_Te_3_@CdS hybrid system for solar‐driven environmental remediation and antibacterial disinfection. On the left, contaminated wastewater containing organic pollutants (e.g., dyes and pesticides) is treated by a floating catalyst composed of Bi_2_Te_3_@CdS coated on PU foam. Under solar irradiation, CdS facilitates photocatalysis, while Bi_2_Te_3_ absorbs NIR light to produce localized heating, accelerating degradation reactions by generating different ROS (•O_2_^−^, •OH, and H_2_O_2_). This synergistic process leads to the mineralization of pollutants into CO_2_ and H_2_O. On the right, a hybrid coating is applied to transparent surfaces (e.g., windows), where sunlight induces a photothermal response (ΔT) that raises surface temperature across the window, leading to bactericidal levels. This enables the effective passive disinfection of pathogens, such as *E. coli* and *S. aureus*, even under ambient light. Overall, this integrated system provides a platform for simultaneous wastewater treatment and room disinfection.

**Figure 2:**
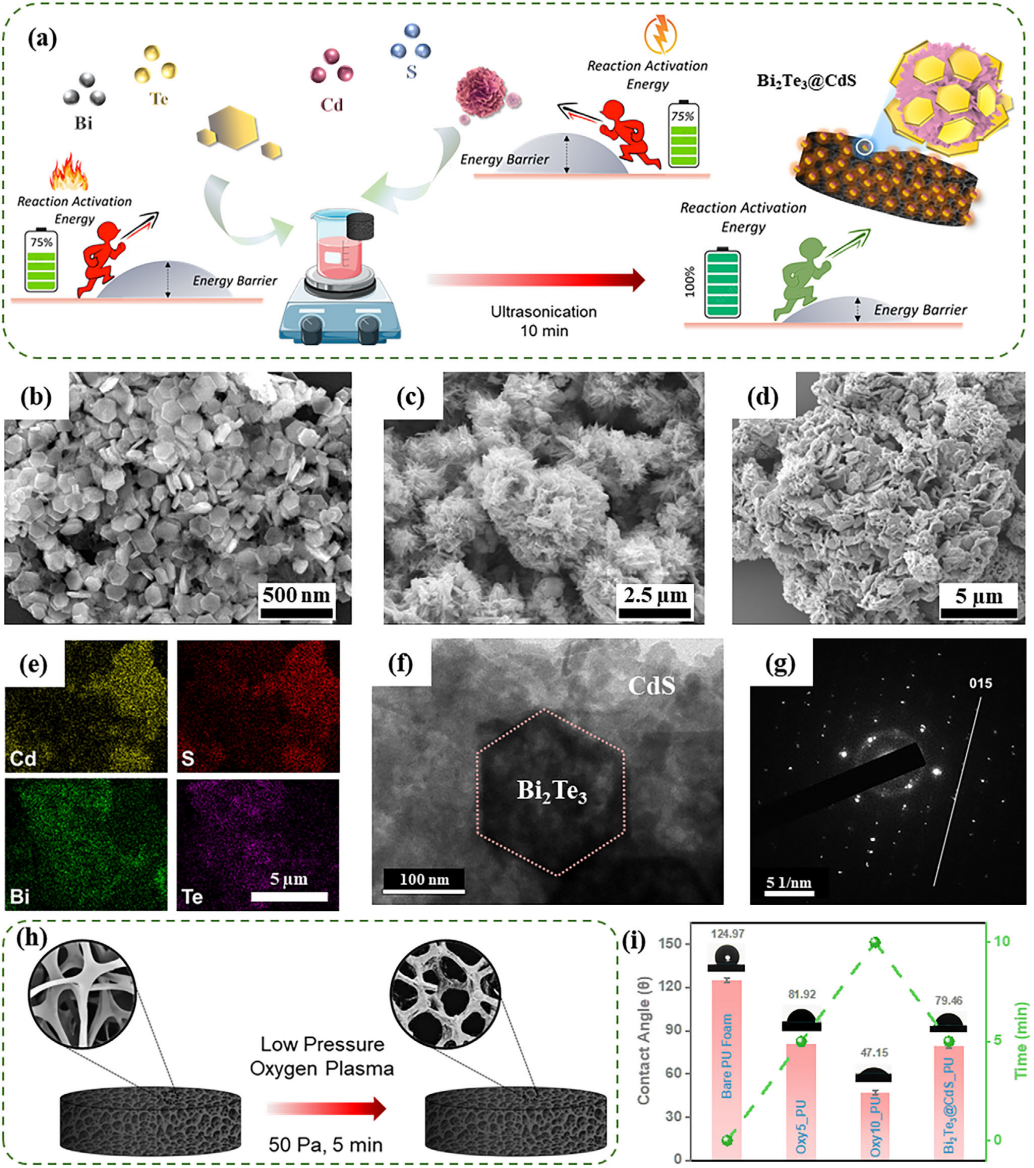
a) Schematic illustration of Bi_2_Te_3_@CdS hybrid synthesis. b–d) SEM image showing the morphology of Bi_2_Te_3_, CdS, and Bi_2_Te_3_@CdS hybrid, respectively. e) EDX mapping of Bi_2_Te_3_@CdS hybrid. f) TEM image of the heterojunction interface between Bi_2_Te_3_ and CdS nanoparticle. g) SAED pattern demonstrating the crystallinity of Bi_2_Te_3_@CdS hybrid. h) Schematic showing increased porosity of PU foam after O_2_ plasma treatment. i) Contact angle measurements of PU foam after plasma and catalyst coating.

Synthesis of Bi_2_Te_3_@CdS hybrid is illustrated in **Figure** **2**a, where individually prepared Bi_2_Te_3_ and CdS nanoparticles were homogeneously mixed to form a hybrid. This hybrid material was subsequently deposited onto oxygen plasma‐treated PU foam, resulting in a uniform and stable coating for further catalytic applications. The morphological studies of individual nanoparticles and the hybrid material were analyzed using scanning electron microscopy (SEM). As shown in Figure 2b, Bi_2_Te_3_ NPs exhibited well‐defined hexagonal nanoplates with lateral dimensions ranging from 300 to 600 nm (Figure S4, Supporting Information). In contrast, Figure 2c presents the morphology of CdS, which formed hierarchical flowerlike structures. These microflowers ranged in diameter from 2 to 3 µm, offering a high surface area conducive to enhanced catalytic interactions (Figure S4, Supporting Information). The elemental composition and morphology of both nanoparticles were analyzed using SEM coupled with Energy‐Dispersive X‐ray Spectroscopy (EDX). EDX spectra confirmed the presence of Bi and Te, indicating formation of Bi_2_Te_3_, and Cd and S, confirming the presence of CdS (Figures S1 and S2, Supporting Information). SEM analysis further revealed that Bi_2_Te_3_ NPs exhibit an average particle size of ≈280 nm, while CdS microflowers possess a larger size, with individual structures measuring ≈2 µm in diameter (Figure S3, Supporting Information). Upon hybridization, SEM images in Figure 2d confirmed successful deposition of Bi_2_Te_3_ NPs onto the surface of CdS microflowers, resulting in a hierarchical architecture. To confirm elemental composition and verify successful hybrid formation, EDX was performed on Bi_2_Te_3_@CdS hybrid. Figure 2e shows the corresponding EDX spectrum, which clearly identifies the presence of Bi, Te, Cd, and S, thus confirming the coexistence of both Bi_2_Te_3_ and CdS phases in the hybrid. Transmission electron microscopy (TEM) analysis in Figure 2f further revealed the interfacial structure of Bi_2_Te_3_@CdS hybrid, demonstrating that distinct Bi_2_Te_3_ adhered to CdS framework, which supports the SEM observations. High‐resolution TEM (HRTEM), as shown in Figure 2g, displayed clear lattice fringes corresponding to both CdS and Bi_2_Te_3_, confirming their crystalline nature and the formation of heterojunctions. The observed SAED patterns of the hybrid confirm the presence of (015) plane, attributing the presence of Bi_2_Te_3_.

To ensure an adhesive and uniform coating of catalyst on PU foam, the foam substrate was pretreated with oxygen plasma at 50 Pa for 5 min, as schematically illustrated in Figure 2h. This surface modification increased surface roughness, thereby enhancing the adhesion properties of the foam.^[^ ^47^, ^48^ ^]^ The effectiveness of this treatment was assessed via contact angle measurements (Figure 2i). The bare PU foam exhibited a high contact angle of 124.97°, indicating hydrophobicity. Following 10 min of plasma treatment, PU foam exhibited significant structural damage, and the water contact angle decreased sharply to 47.15°, indicating excessive surface modification that may compromise material integrity. In contrast, 5‐min plasma exposure led to a substantial reduction in contact angle to 81.92°, demonstrating improved surface hydrophilicity without noticeable material degradation. Furthermore, a 5 min plasma‐treated PU foam coated with Bi_2_Te_3_@CdS hybrid displayed a slightly reduced contact angle of 79.46°, confirming the successful deposition of the hybrid material onto the treated PU surface. These comprehensive morphological and structural analyses underscore the importance of surface engineering and hierarchical structuring in achieving uniform hybrid material integration onto 3D scaffold, setting the stage for improved catalytic performance.

### 2.2 Chemical Characterizations of Bi_2_Te_3_@CdS Hybrid

**Figure 3:**
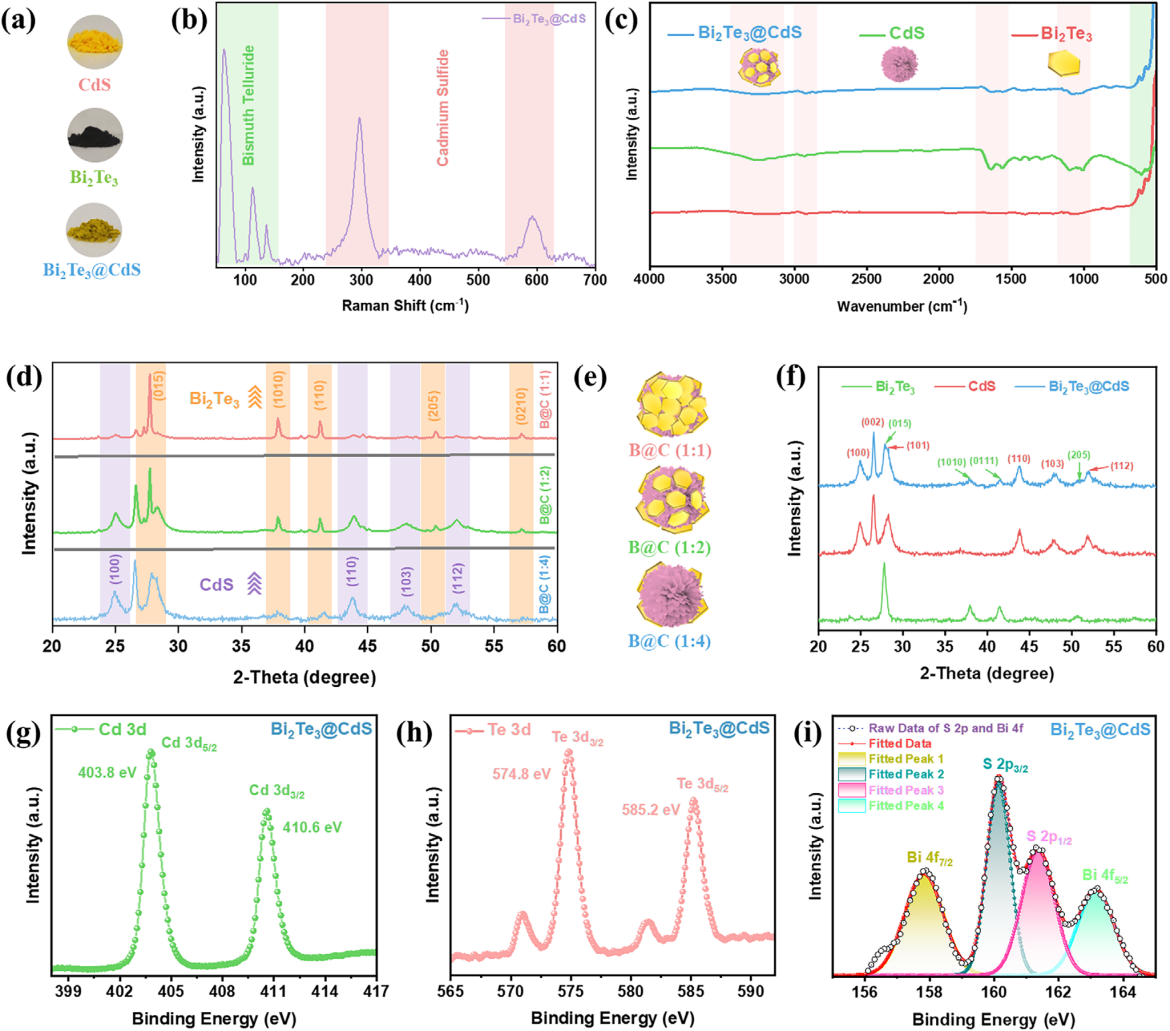
a) Photographs of synthesized CdS, Bi_2_Te_3_, and Bi_2_Te_3_@CdS hybrid powders. b) Raman spectrum of Bi_2_Te_3_@CdS hybrid. c) FTIR spectra showing functional groups present in CdS, Bi_2_Te_3_, and Bi_2_Te_3_@CdS hybrid. d,e) XRD patterns and Schematic illustration of Bi_2_Te_3_@CdS hybrids with varying ratios. f) XRD patterns comparing Bi_2_Te_3_, CdS, and Bi_2_Te_3_@CdS hybrid. g,h) XPS spectrum of Cd 3d region and Te 3d region in Bi_2_Te_3_@CdS hybrid. i) High‐resolution XPS spectra showing Bi 4f and S 2p peaks in Bi_2_Te_3_@CdS hybrid.

**Figure** **3** comprehensively characterizes the chemical properties of Bi_2_Te_3_@CdS hybrid. Figure 3a presents the optical appearance of individual and hybrid materials, where CdS appears yellow, Bi_2_Te_3_ is black, and their composite exhibits a dark yellow hue, indicating successful hybridization of both components. Raman spectrum in Figure 3b reveals characteristic vibrational modes of Bi_2_Te_3_ (highlighted in green‐shaded region, < 200 cm^−1^) and CdS (red‐shaded region, ≈300 and ≈600 cm^−1^).^[^ ^49^, ^50^, ^51^ ^]^ Thereby confirming the successful formation of the hybrid structure without inducing notable structural disruption in either phase. For the pristine materials, all the corresponding Raman peaks are distinctly observed in the individual spectra of both Bi_2_Te_3_ and CdS nanoparticles (Figure S5, Supporting Information). Fourier‐transform infrared (FTIR) spectroscopy (Figure 3c) provides additional evidence of successful hybrid formation. Bi_2_Te_3_@CdS hybrid spectrum retains the characteristic vibrational features of both CdS and Bi_2_Te_3_, indicating the successful integration of both phases.^[^ ^52^, ^53^, ^54^ ^]^ To optimize the composition of Bi_2_Te_3_@CdS hybrid, different weight ratios of Bi_2_Te_3_ to CdS (1:1, 1:2, and 1:4) were prepared. X‐ray diffraction (XRD) analyses revealed that increasing Bi_2_Te_3_ content in the hybrid system (at 1:1 ratio) results in a corresponding intensification of its characteristic diffraction peaks (Figure 3d). Based on this observation, a 1:2 ratio was selected for subsequent experiments, as it offers a balanced contribution from both Bi_2_Te_3_ and CdS. This composition is designed to maximize solar light utilization, allowing both components to contribute effectively. In contrast, a 1:1 ratio with excessive Bi_2_Te_3_ coverage may hinder the exposure of CdS to sunlight, thereby limiting its photoactivity (Figure 3e). The phase purity and crystallinity of the optimized hybrid are further corroborated by XRD patterns (Figure 3f), where Bi_2_Te_3_@CdS sample exhibits all major diffraction peaks of both Bi_2_Te_3_ and CdS, without any extraneous peaks.^[^ ^55^, ^56^ ^]^ This confirms the preservation of individual crystal structures and the absence of impurity phases. As shown in Figure S6 (Supporting Information), XRD patterns of the as‐synthesized Bi_2_Te_3_ and CdS nanoparticles exhibit diffraction peaks that match well with the standard JCPDS cards (15‐0863) and (892944), respectively.

X‐ray photoelectron spectroscopy (XPS) analysis further elucidates the chemical states of the constituent elements. The high‐resolution Cd 3d spectrum (Figure 3g) reveals two prominent peaks at 403.8 eV (Cd 3d_5/2_) and 410.6 eV (Cd 3d_3/2_), characteristic of Cd^2+^ in CdS, confirming the chemical integrity of CdS phase within the hybrid. Similarly, Te 3d spectrum (Figure 3h) shows peaks at 574.8 eV (Te 3d_5/2_) and 585.2 eV (Te 3d_3/2_), consistent with Te environment in Bi_2_Te_3_, indicating the preservation of Bi_2_Te_3_ phase. Finally, the deconvoluted XPS spectra of S 2p and Bi 4f regions (Figure 3i) display well‐resolved peaks for S 2p_3/2_ and S 2p_1/2_, as well as Bi 4f_7/2_ and Bi 4f_5/2_, confirming the presence and chemical interaction of both sulfide and bismuth species at the interface. The fitting of multiple peaks confirms the formation of a well‐defined heterojunction, characterized by strong interfacial coupling between Bi_2_Te_3_ and CdS. Figure S7 (Supporting Information) shows the full survey spectrum of all three materials. For Bi_2_Te_3_, Bi 4f spectrum displays two prominent peaks at 157.5 and 162.8 eV, attributed to Bi 4f_7/2_ and Bi 4f_5/2_, while Te 3d region shows Te 3d_5/2_ and Te 3d_3/2_ peaks at 574.5 and 584.8 eV, along with minor oxidation features.^[^ ^57^ ^]^ In CdS spectra, Cd 3d_5/2_ and Cd 3d_3/2_ are observed at 404.2 and 410.9 eV, respectively, and S 2p exhibits doublets at 160.5 eV (S 2p_3/2_) and 161.8 eV (S 2p_1/2_) (Figure S8, Supporting Information).^[^ ^58^ ^]^ The observed shift toward lower binding energies in Cd 3d_5/2_ and Cd 3d_3/2_ peaks in the XPS spectrum of the hybrid indicates an increased electron density around Cd atoms. This electronic modulation is further corroborated by charge density plots obtained from DFT simulations (Section 2.4), which reveal electron accumulation at Cd sites within the heterostructure. Collectively, these results provide robust evidence for the successful synthesis of Bi_2_Te_3_@CdS hybrid nanostructures with high crystallinity and interfacial contact.

### 2.3 Optical and Electrical Characterizations

**Figure 4:**
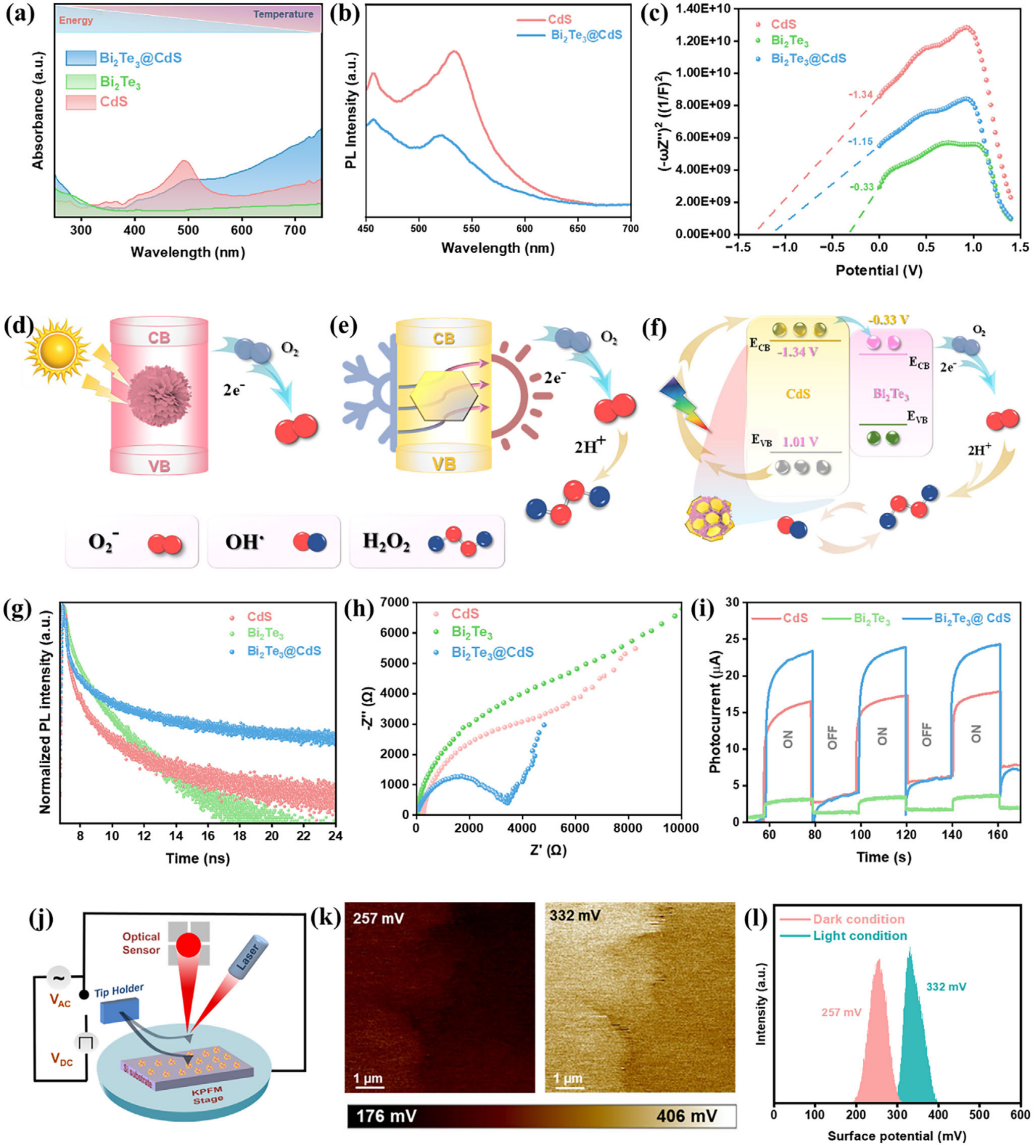
a,b) UV–vis absorption and PL spectra of CdS, Bi_2_Te_3_, and Bi_2_Te_3_@CdS hybrid. c) Mott‐Schottky plots of CdS, Bi_2_Te_3_, and Bi_2_Te_3_@CdS hybrid. d,e) Schematic illustration of ROS generation from CdS under light irradiation and Bi_2_Te_3_ under thermal gradient. f) Proposed charge transfer mechanism and energy band alignment for Bi_2_Te_3_@CdS heterojunction for enhanced ROS generation (Potential vs RHE). g) TRPL decay curves revealing charge carrier lifetimes. h) EIS Nyquist plots showing charge transfer resistance. i) Photocurrent responses of CdS, Bi_2_Te_3_, and Bi_2_Te_3_@CdS hybrid. j) Schematic diagram of KPFM setup used to measure surface potential. k,l) KPFM surface potential images and distribution of Bi_2_Te_3_@CdS under the light and dark conditions.

Figure S9 (Supporting Information) shows the UV–Vis–NIR absorbance spectrum (200–1400 nm) of Bi_2_Te_3_@CdS hybrid, demonstrating its broadband light absorption capability, which spans from UV to NIR region. This extended spectral response is primarily attributed to Bi_2_Te_3_’s strong NIR absorption and photothermal conversion, complementing CdS's efficient visible light absorption. In **Figure** **4**a, a comparative UV–vis absorbance analysis of Bi_2_Te_3_, CdS, and Bi_2_Te_3_@CdS hybrid reveals that the hybrid exhibits significantly enhanced and broadened light absorption relative to either component alone. This synergistic behavior confirms that the integration of Bi_2_Te_3_ and CdS facilitates complementary spectral coverage, enhancing solar energy harvesting efficiency across the solar spectrum. Tauc plot derived from UV–vis spectroscopy and Diffuse Reflectance Infrared Fourier Transform (DRIFT) data illustrates the bandgap energies of Bi_2_Te_3_, CdS, and Bi_2_Te_3_@CdS hybrid (Figure S10, Supporting Information). Bi_2_Te_3_ exhibits a bandgap of 0.27 eV, determined via DRIFT, reflecting its strong near‐IR absorption. CdS exhibits a bandgap of 2.35 eV, characteristic of its visible light absorption, as determined by UV–vis spectroscopy. Bi_2_Te_3_@CdS hybrid, also analyzed via UV–vis, displays an intermediate bandgap of 2.23 eV, indicating enhanced light harvesting.

In Figure 4b, pristine CdS exhibits strong PL emission, which is significantly quenched upon hybridization with Bi_2_Te_3_. Bare CdS exhibits high PL intensity at ≈460 nm, indicating a high rate of radiative recombination of charge carriers. This means that a significant number of photoexcited charge carriers recombine quickly, emitting photons, which results in a strong PL signal intensity. Upon hybridization, the lower PL intensity decreased significantly, suggesting that charge carriers are separated more effectively or transferred to Bi_2_Te_3_, which suppresses radiative recombination. The reduced recombination means that the charge carriers persist longer in their excited states, thereby increasing their chances of participating in surface reactions rather than simply emitting light.^[^ ^59^ ^]^ The appearance of a new peak at 526 nm in Bi_2_Te_3_@CdS is attributed to the presence of surface defect state emissions, possibly due to the formation of CdS nanoflower structure. Correspondingly, in Figure 4g, time‐resolved PL decay is performed using a 405 nm laser, which shows that the hybrid has a longer‐lived carrier population than pristine CdS, indicating that recombination is slowed. Bi_2_Te_3_ exhibits a short average lifetime (1.263 ns), indicative of rapid charge recombination, while CdS shows a longer lifetime (4.231 ns) due to more efficient charge separation. Notably, Bi_2_Te_3_@CdS hybrid achieves the longest lifetime (7.947 ns), demonstrating effective suppression of recombination and enhanced carrier separation at the heterojunction (Table S1, Supporting Information). Mott‐Schottky analysis (Figure 4c) provides the flat‐band potential of each material. CdS, which is known as an n‐type semiconductor, shows much more negative flat‐band potential (≈−1.34 eV vs. RHE) than Bi_2_Te_3_, which is semi‐metallic in nature (≈−0.33 eV). The hybrid catalyst exhibited a more negative conduction band potential, demonstrating its enhanced ability to reduce oxygen. Figure 4d illustrates the schematic pathways for the stepwise reduction of molecular O_2_ to •O_2_^−^ radical for CdS alone, and Figure 4e further shows how hot electrons from Bi_2_Te_3_ reducing O_2_ to •O_2_^−^ radical, which then converts into H_2_O_2_. Both Bi_2_Te_3_ and CdS possess conduction band potentials more negative than ‐0.3 eV versus RHE, rendering them thermodynamically favorable for driving O_2_ reduction reactions, which are essential for ROS generation.^[^ ^60^, ^61^ ^]^ The schematic band diagram (Figure 4f) thus reflects a heterojunction where upon contact, electrons energetically flow from CdS to Bi_2_Te_3_. This built‐in field at the interface drives the spatial separation of carriers. The measured flat‐band potentials (via Mott‐Schottky) agree with this picture and verify that the band edges straddle potentials for O_2_ reduction into all ROS (•O_2_^−^, •OH, and H_2_O_2_).

Electrochemical impedance spectroscopy (EIS, Figure 4h) and transient photocurrent measurements (Figure 4i) provide insights into the charge carrier dynamics of materials. The Nyquist plot in Figure 4h reveals that Bi_2_Te_3_@CdS hybrid exhibits a significantly smaller semicircular diameter compared to pristine CdS and Bi_2_Te_3_, indicating a lower charge‐transfer resistance at the interface and faster interfacial charge transport kinetics. This reduction in impedance suggests more efficient separation and transfer of photogenerated carriers across the heterojunction interface. Complementarily, the photocurrent response shown in Figure 4i demonstrates that Bi_2_Te_3_@CdS hybrid generates a substantially higher and more stable photocurrent under chopped light illumination than either of its individual components. The enhanced on‐off photocurrent behavior directly indicates that a greater number of photoexcited charge carriers are successfully reaching the external circuit rather than undergoing recombination. These improvements in both impedance and photocurrent responses affirm that the heterostructure facilitates efficient charge separation and rapid carrier transport.

Figure 4j shows the setup for mapping the surface potential of materials, providing direct evidence of built‐in fields and voltage. Kelvin probe force microscopy (KPFM) images reveal that Bi_2_Te_3_@CdS surface exhibits a distinct voltage contrast in the light condition as compared to the dark condition, indicating charge redistribution at the heterointerface (Figure 4k). The Gaussian histograms of surface potential (Figure 4l) further show that under illumination, the hybrid's surface potential shifts (from 257 to 332 mV) relative to the dark. This light‐induced modulation of surface potential indicates a reduced work function, which facilitates enhanced charge transfer and improves the catalyst's participation in the photoreaction process.^[^ ^62^ ^]^ In general, KPFM measures the contact potential difference and can visualize charge flow and distribution.^[^ ^63^ ^]^ Indeed, it is well recognized that PL quenching and KPFM shifts are complementary signatures of interfacial charge transfer.^[^ ^64^, ^65^ ^]^ Thus, Figure 4b,l demonstrates that Bi_2_Te_3_@CdS junction establishes a persistent internal field that helps to maintain charge separation, which is critical for generating ROS.

Together, the above characterizations paint a picture of synergistic carrier dynamics in the hybrid catalyst. Under solar illumination, CdS absorbs visible photons and generates electron‐hole pairs, while Bi_2_Te_3_ absorbs longer‐wavelength light (NIR component of the solar spectrum) and converts it to heat. The thermal energy in Bi_2_Te_3_ has two effects: first, it directly excites carriers in this narrow‐gap semiconductor, and second, it raises the local temperature to accelerate reaction kinetics. Also, recent studies have shown that thermoelectric materials like Bi_2_Te_3_ under a temperature gradient can produce ROS such as •O_2_^−^ and H_2_O_2_.^[^ ^30^ ^]^ Bi_2_Te_3_ NPs alone were demonstrated to generate H_2_O_2_ and kill bacteria when subjected to a small ΔT (through Seebeck‐driven electron‐hole separation).^[^ ^33^ ^]^ In our hybrid system, photothermal heating of Bi_2_Te_3_ acts in concert with CdS photoexcitation. Photogenerated electrons in CdS flow into Bi_2_Te_3,_ and hot electrons thermally excited in Bi_2_Te_3_ can participate in ROS generation for catalytic applications.

### 2.4 Density Functional Theory (DFT) Simulations

**Figure 5:**
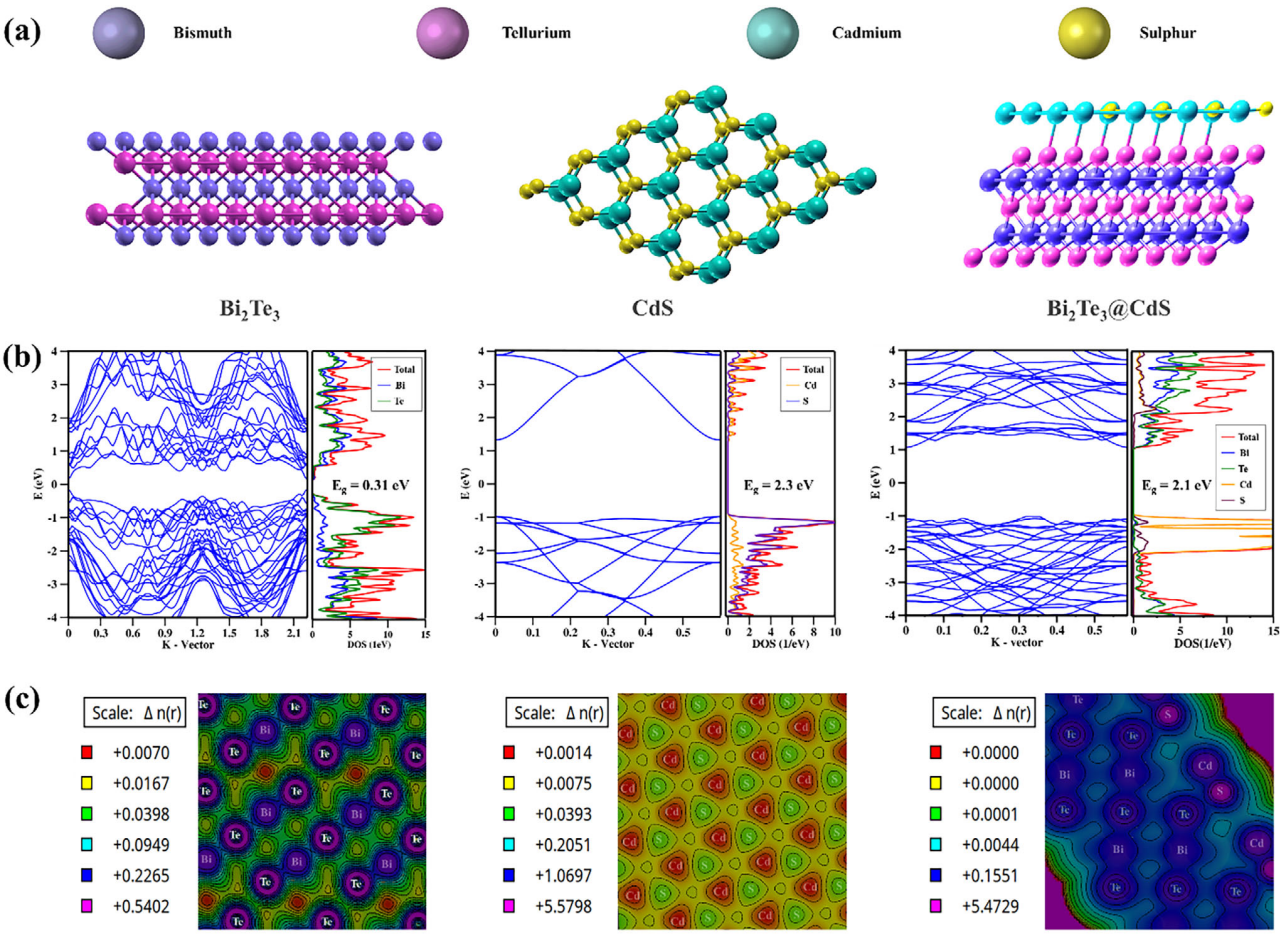
a) Atomic structures of Bi_2_Te_3_, CdS, and Bi_2_Te_3_@CdS hybrid. b) Electronic band structures and DOS for Bi_2_Te_3_, CdS, and Bi_2_Te_3_@CdS hybrid. c) Charge density difference maps illustrating electron distribution in Bi_2_Te_3_, CdS, and Bi_2_Te_3_@CdS hybrid.

To gain a comprehensive understanding of the electronic structure and interfacial charge transfer characteristics of Bi_2_Te_3_@CdS heterojunction, DFT simulations were conducted. As shown in **Figure** **5**a, the optimized atomic configurations of Bi_2_Te_3_, CdS, and Bi_2_Te_3_@CdS hybrid illustrate the distinct lattice geometries of each material. Bi_2_Te_3_ adopts a layered rhombohedral structure (space group R3̅m), characterized by quintuple layers of alternating Bi and Te atoms held together by van der Waals interactions, while CdS exhibits a hexagonal wurtzite‐type structure composed of tetrahedrally coordinated Cd and S atoms. The calculated band structures and projected density of states (DOS) for each system are presented in Figure 5b. Bi_2_Te_3_ exhibits a bandgap of 0.31 eV, which aligns well with its known semi‐metallic behavior and matches closely with our experimentally determined value of 0.27 eV. DOS shows significant contributions from Bi and Te p‐orbitals near the Fermi level, indicating the active role of these atoms in charge transport. CdS, on the other hand, reveals a direct bandgap of 2.3 eV, in good agreement with the experimental bandgap of 2.35 eV.

Remarkably, Bi_2_Te_3_@CdS hybrid exhibits a reduced bandgap of 2.1 eV in DFT simulations, corroborating our experimental observation of a bandgap narrowing to 2.23 eV. This redshift in the optical absorption edge is indicative of favorable electronic coupling between Bi_2_Te_3_ and CdS domains. Hybrid's DOS profile shows overlapping contributions from Bi, Te, Cd, and S atoms near the Fermi level, reflecting strong interfacial orbital hybridization. Further insights into the nature of charge redistribution at the interface are obtained from charge density difference plots (Δn(r)), depicted in Figure 5c. For pristine Bi_2_Te_3_, electron accumulation is predominantly localized around Te atoms, consistent with their higher electronegativity and role as electron‐rich centers. In contrast, Bi atoms exhibit slight depletion, attributed to their electron‐donating 6s2 lone pairs. In CdS, the electron density is concentrated around S atoms, with a corresponding depletion at Cd sites. In Bi_2_Te_3_@CdS hybrid, the interface displays a pronounced charge redistribution, characterized by strong electron accumulation at Te‐S and Bi‐Cd contact regions, indicating robust electronic coupling. These interfacial charge polarization regions suggest the formation of an internal electric field at the heterojunction, which facilitates spatial separation of photogenerated charge carriers and suppresses recombination.

DFT‐calculated total density of states (TDOS) plots for all three materials reveal distinct electronic structures (Figure S11, Supporting Information). Bi_2_Te_3_ shows a high DOS near the Fermi level, consistent with its semi‐metallic nature, while CdS displays a clear bandgap characteristic of a semiconductor. In Bi_2_Te_3_@CdS hybrid, TDOS features significant overlap among Bi, Te, Cd, and S contributions near the Fermi level, indicating strong interfacial orbital hybridization. This electronic coupling leads to bandgap narrowing and enhanced charge delocalization, supporting efficient charge separation and underpinning the hybrid's superior visible‐NIR catalytic performance. Taken together, these DFT results provide compelling evidence that the formation of Bi_2_Te_3_@CdS heterojunction induces synergistic modifications to the band structure, enhances charge delocalization, and promotes efficient carrier separation. These electronic features are consistent with the experimentally observed bandgap and form the foundation for the hybrid's improved performance in Vis‐NIR‐driven catalytic applications.

### 2.5 Photothermal Properties and ROS Generation

**Figure 6:**
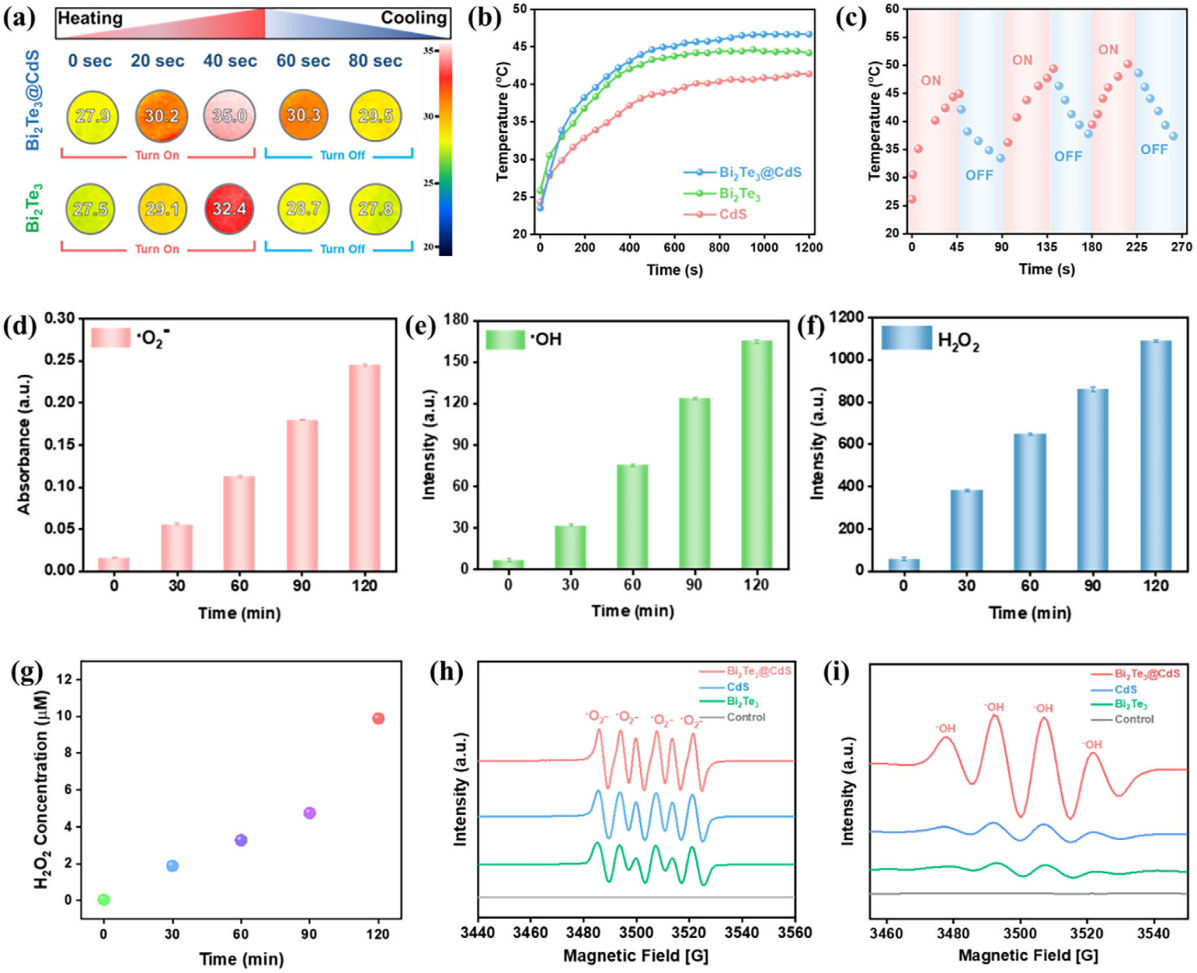
a) IR imaging for temperature mapping images showing the heating and cooling process of Bi_2_Te_3_@CdS and Bi_2_Te_3_. b) Time‐dependent temperature profiles of Bi_2_Te_3,_ CdS, Bi_2_Te_3_@CdS. c) Reversible temperature switching behavior during repeated ON/OFF irradiation cycles. d) Generation of •O_2_^−^ over time, measured by absorbance. e,f) Time‐dependent generation •OH and H_2_O_2,_ respectively. g) Quantitative analysis of H_2_O_2_ concentration. h,i) EPR spectra of •O_2_^−^ and •OH radicals for different samples.

Infrared (IR) thermal images (**Figure** **6**a) reveal that Bi_2_Te_3_@CdS‐coated PU foam heats much more than Bi_2_Te_3_ alone under 100 mW cm^−2^ illumination. Bi_2_Te_3_@CdS reached a significantly higher surface temperature (35 °C) in 40 s of light exposure compared to pure Bi_2_Te_3_ (32.4 °C) and cooled back more slowly. This rapid heating is consistent with the known broadband absorption and high photothermal conversion of Bi_2_Te_3_ NPs. Here, the hybrid's greater peak temperature and slower cooling imply enhanced nonradiative relaxation (photothermal) pathways, likely due to the combined absorbance of CdS (visible light) and Bi_2_Te_3_ (broadband). Figure S13 (Supporting Information) presents a single heating and cooling cycle, demonstrating that the hybrid exhibits superior heat retention capabilities compared to pristine Bi_2_Te_3_. The higher temperature for the hybrid indicates it can harvest more solar energy as heat, which is advantageous for thermocatalysis. Time‐dependent temperature rises in aqueous dispersions (Figure 6b) confirm the same trend. Bi_2_Te_3_@CdS warms water faster and to a higher steady‐state temperature than either of its components. Over 1200 s of solar simulation, the hybrid dispersion's temperature climbs steadily above those of pure Bi_2_Te_3_ or CdS. For instance, Bi_2_Te_3_ alone raises the solution by ≈15–20 °C and CdS by only ≈5–15 °C, the hybrid pushes it closer to ≈25°C. This enhanced heating reflects the sum of both materials’ absorption plus synergistic thermalization. The continuous heating indicates efficient light harvesting and stable photothermal conversion.

Figure 6c plots continuous on‐off illumination cycles (solar on/off) for Bi_2_Te_3_@CdS foam. The maximum temperature reached in every 45 s “on” cycle is nearly identical (≈45 °C on the first cycle, with an increment to 50 °C by the third cycle), demonstrating good photothermal stability. This reproducible cycling indicates that Bi_2_Te_3_@CdS hybrid does not degrade or change its thermal properties over multiple solar exposures. Such stability is critical for practical solar‑thermal applications, which further confirms that the heat generation mechanism is inherent and reversible. Upon exposure to solar irradiation, Bi_2_Te_3_@CdS heterostructure efficiently ROS, as demonstrated in (Figure 6d–f). Figure 6d shows •O_2_^−^ generation measured by the XTT assay, where the hybrid's curve rises steeply over 120 min, accumulating generated •O_2_^−^ concentration. Similarly, Figure 6e illustrates •OH radical generation assessed via terephthalic acid fluorescence, where an increase in fluorescence intensity over time indicates efficient •OH production. In Figure 6f, H_2_O_2_ generation is quantified using the Amplex Red assay, which confirms the formation of H_2_O_2_ through conversion of •O_2_^−^, as described by reaction (1).^[^ ^12^, ^66^ ^]^ The enhancement in all ROS generation is attributed to the efficient charge separation facilitated by the heterojunction structure. Photogenerated electrons preferentially reduce molecular oxygen (O_2_) to •O_2_^−^, which subsequently converts to H_2_O_2_, and further to •OH via UV‐induced photolysis (reaction 2).^[^ ^67^, ^68^ ^]^

(1) $$\cdot {O_{2}}^{-}+e^{-}+2H^{+}\to H_{2}O_{2}$$

(2) H2O2+U.V→2•OH

Heterojunctions are well known to boost interfacial charge transfer and ROS production. In contrast, CdS alone cannot utilize the full spectrum and also suffers from rapid recombination, so it generates minimal ROS only. Figure 6g illustrates the specific H_2_O_2_ yield of the hybrid over a 120 min period, during which ≈10 µmol of H_2_O_2_ is generated. Notably, the yield rate is reflected by the nearly constant slope, which remains stable throughout the experiment. These findings demonstrate that the conduction band of Bi_2_Te_3_@CdS hybrid is well‐suited for O_2_ reduction, and its photogenerated charge carriers persist long enough to generate all three ROS efficiently. Electron paramagnetic resonance (EPR) spin‐trap spectra provide direct evidence of free radicals. Figure 6h shows the characteristic DMPO‐•O_2_^−^ adduct peaks, and Figure 6i shows DMPO‐•OH. In both spectra, Bi_2_Te_3_@CdS exhibits the strongest signal intensities (highest peak amplitudes) compared to Bi_2_Te_3_ or CdS alone. This confirms that the hybrid generates the highest concentrations of •O_2_^−^ and •OH. These EPR results corroborate that Bi_2_Te_3_@CdS supports the most efficient ROS formation. Finally, EPR confirms that the hybrid interface generates abundant charge‐driven radicals, whereas the single components fall far behind.

### 2.6 Wastewater Treatment

**Figure 7:**
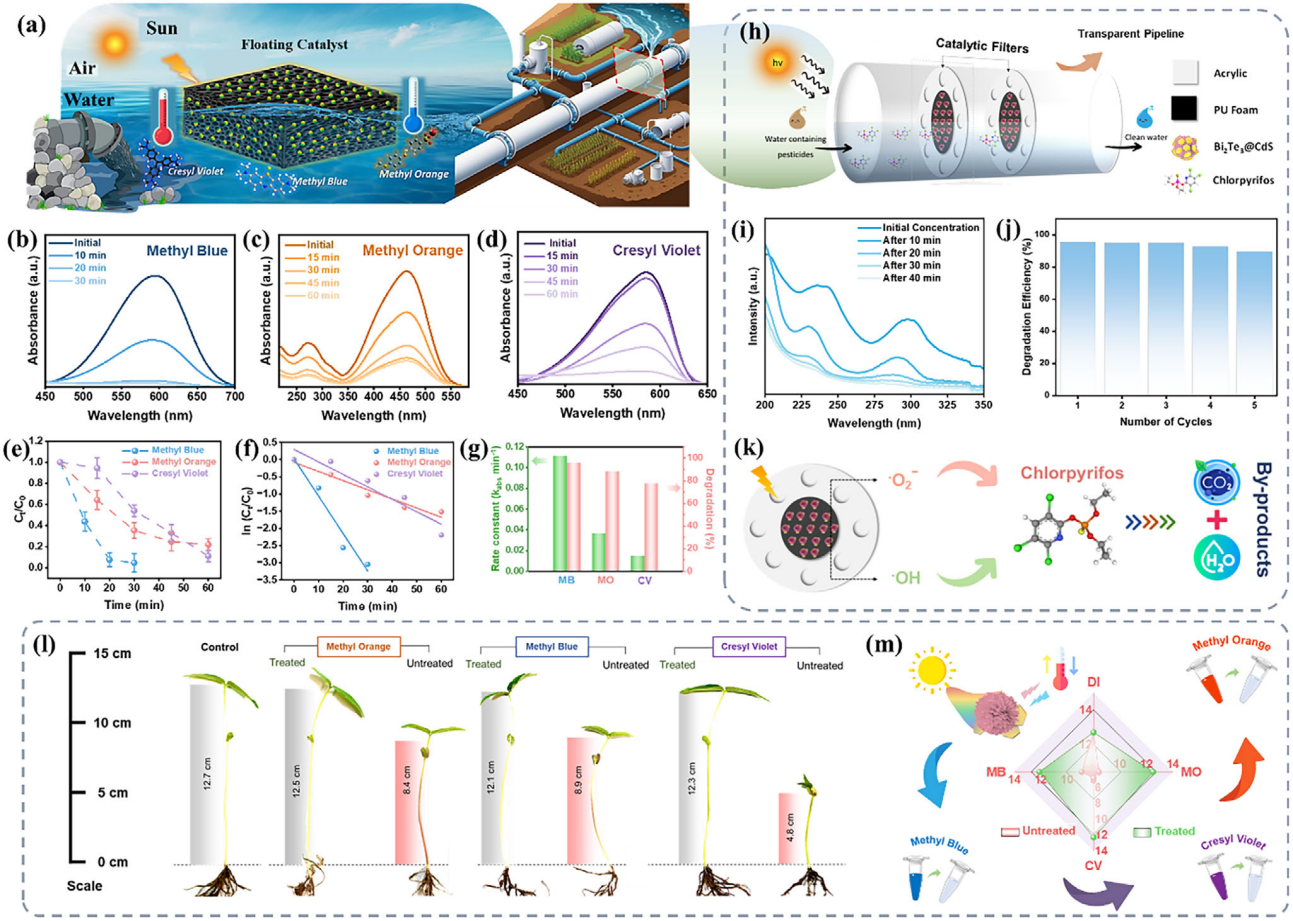
a) Schematic illustration of a catalytic water treatment system using Bi_2_Te_3_@CdS‐based filters for dye degradation. b–d) Absorbance spectra showing the degradation of MB, MO, and CV over time, respectively. e) Plot of relative concentration (C_t_/C_0_) versus time for the catalytic degradation of three dyes. f) Kinetic analysis (ln(C_t_/C_0_) vs. time). g) Bar graph comparing the removal efficiency and rate constants. h) Schematic of the catalytic filter setup for continuous flow treatment. i) UV–vis spectra showing degradation of CPS. j) Bar chart showing the degradation efficiency over multiple cycles. k) Mechanism illustration of catalytic degradation of CPS into harmless by‐products. l) Photographs and measurements of Moong Bean plants' growth using water containing different dyes, and after catalysis treatment. m) Radar chart comparing the overall growth of plants (unit in cm).

**Figure** **7**a depicts the conceptual architecture of Bi_2_Te_3_@CdS‐coated PU foam, designed as a floating photothermocatalytic platform for solar‐driven water remediation. When deployed in open‐water environments or flow‐through conduits, the ultralight PU foam remains buoyant at the air‐water interface, ensuring continuous exposure of the catalyst layer to sunlight and atmospheric oxygen (ambient air). Under illumination, foam's hydrophilic porous network establishes a vertical temperature gradient that drives light‐induced convective flow, a “self‐pumping” transport enhancement in floating PU‐based photoreactors, thereby improving the delivery of pollutants and dissolved oxygen to active sites. Bi_2_Te_3_ component, with its broad visible‐to‐NIR absorption, efficiently converts incident photons into localized heat, increasing the microenvironment temperature, whereas CdS provides strong visible‐light absorption and active photocatalytic centers. The formation of Bi_2_Te_3_@CdS heterojunction facilitates directional charge separation and suppresses electron‐hole recombination, while the photothermally elevated temperature accelerates reaction kinetics and boosts the generation of reactive oxygen species. Through the integration of physical adsorption, photothermal heating, and thermocatalytic/photocatalytic activity within a single floating architecture, Bi_2_Te_3_@CdS‐coated PU foam functions as a highly efficient, self‐regulating remediation platform capable of enhanced mass transport and intensified solar‐driven catalysis.

#### 2.6.1 Degradation of Industrial Dyes (MB, MO, CV)

Under simulated solar irradiation (AM 1.5G, 100 mW cm^−2^), Bi_2_Te_3_@CdS foam efficiently degrades all three model dyes (10 mg/l). The time‐dependent UV–vis spectra (Figure 7b–d) show the main absorbance peaks of Methyl Blue (MB), Methyl Orange (MO), and Cresyl Violet (CV) steadily diminishing. In Figure 6d,f, a clear time‐dependent increase in the concentrations of both ROS species (•O_2_^−^ and •OH) is observed, indicating progressively enhanced generation of reactive radicals under illumination. This continuous rise in ROS production reflects the sustained activation of the catalyst surface and provides direct evidence that more oxidative species participate in the degradation pathway as the reaction proceeds. The higher availability of •O_2_^−^ and •OH accelerates the breakdown of dye molecules, thereby explaining the improved degradation efficiency over time. In 30 min, MB's characteristic peak nearly vanishes, indicating ≈97% removal, whereas MO and CV require 60 min to reach comparable levels. Figure 7e illustrates the degradation profiles of all dyes, showing that MB undergoes the most rapid and extensive removal, achieving near‐complete degradation within 30 min, whereas MO and CV exhibit slower degradation rates. These values correspond to ≈97%, 93%, and 88% degradation. The data fit well with pseudo‐first‐order kinetics (‐ln C_t_/C_0_ vs. time), yielding apparent rate constants k of ≈0.12, 0.034, and 0.015 min^−1^ for MB, MO, and CV, respectively (Figure 6f). Detailed kinetic parameters were extracted from the data (Figure 6g). For MB, k ≈ 0.11 min^−1^ and ≈97% degradation in 30 min, for MO, k ≈ 0.034 min^−1^ and ≈93% in 60 min, for CV, k ≈ 0.015 min^−1^ and ≈88% in 60 min. These kinetic constants indicate MB degrades ≈3× faster than MO or CV. The superior MB performance may stem from its higher molar absorptivity and easier oxidation. Importantly, all dyes showed greater than 80% removal within 1 h.

To confirm the synergistic photothermal effect of Bi_2_Te_3_@CdS to thermal treatment alone. Figure S14 (Supporting Information) presents the photothermal and thermal catalytic degradation performance of Bi_2_Te_3_@CdS hybrid for MB dye (10 mg/l). Under simulated sunlight, the characteristic absorbance peak of MB diminishes rapidly, nearly disappearing within 30 min, indicating efficient photothermal‐driven degradation. In contrast, under thermal‐only conditions (60 °C), the MB peak decreases more gradually, requiring 40 min for substantial removal. Photothermal treatment achieves a significantly faster decline in dye concentration, with C_t_/C_0_ approaching zero within 30 min, whereas thermal treatment alone results in slower, incomplete degradation within the same period. The photothermal process yields a higher rate constant (≈0.11 min^−1^) and >95% degradation, whereas the thermal process exhibits a lower rate constant and reduced degradation efficiency. Figure S15 (Supporting Information) shows the effect of incident light intensity on the degradation of (MB, 10 mg/l) using simulated solar irradiation (200 and 100 mW cm^−2^ irradiation). In both cases, the characteristic MB absorbance peak decreases progressively with increasing irradiation time, indicating efficient degradation of the dye. Notably, a higher light intensity (200 mW cm^−2^) results in a more rapid decline in absorbance compared to 100 mW cm^−2^. MB degradation proceeds faster at 200 mW cm^−2^, with C_t_/C_0_ dropping below 0.1 within 30 min, while at 100 mW cm^−2^, a similar removal efficiency is achieved but at a slightly slower rate. These results demonstrate that increasing the incident light intensity significantly enhances the catalytic degradation kinetics, underscoring the importance of photothermal input in driving efficient pollutant removal.

We also tested the removal of chlorpyrifos (10 mg/l) under the same conditions (as above). Figure 7h illustrates the conceptual scheme in which Bi_2_Te_3_@CdS foam, irradiated by light, generates ROS that oxidize water containing harmful pesticides. UV–vis spectra (Figure 7i) of CPS display a characteristic peak which steadily diminishes over 40 min. After this time, CPS is ≈95% degraded. This high degradation efficacy underscores the hybrid catalyst's versatility, where the same ROS (•OH and •O_2_^−^) that decompose dyes also attack the pesticide's bonds. Overall, Bi_2_Te_3_@CdS foam exhibits superior efficacy in the solar‐driven mineralization of CPS, underscoring its significant potential for practical water treatment applications, particularly when benchmarked to previously reported catalytic systems, as detailed in **Table** **1**.

**Table 1:** Comparison of CPS degradation with different catalysts.

Catalysts	Conc. (ppm)	Catalyst Dosage (g/l)	Degradation Time (min)	Efficiency	Refs.
Ni‐Co LDH/Fe_3_O_4_	10	0.025	60	88.1	[69]
CuO/TiO_2_/PANI	5	0.45	90	95	[70]
α‐Bi_2_O_3_ NPs	10	0.1	120	93	[71]
CS/g‐C_3_N_4_	10	2	50	85	[72]
CuS‐Bi_2_O_2_CO_3_	10	0.25	180	95	[73]
MnV_2_O_6_/h‐BN	13	0.06	105	89	[74]
CdS/MAX	20	1	90	93	[75]
Ni_0.9_Mg_0.1_A_l2_O_4_	20	0.5	120	78.83	[76]
ZVI@Al^mix^	10	0.3	360	93.47	[77]
N‐CQDs@ZnHCF	20	1	70	91	[78]
Bi_2_Te_3_@CdS	10	0.1	50	95	This work

Figure 7j assesses the recyclability of the catalyst‐coated foam, which was recovered after each run and reused for CPS degradation five times. CPS degradation efficiency remained very high through all cycles (e.g. ≈95% in cycle 1, ≈90% by cycle 5). The PU foam scaffold contributes to durability by physically trapping the catalyst, and the strong Bi_2_Te_3_@CdS adhesion prevents leaching. Such reusability is essential for practical application, and the observed robustness confirms that the catalytic mechanism does not irreversibly damage the material over multiple uses. To elucidate the CPS degradation mechanism, we propose the pathway sketched in Figure 7k.^[^ ^79^, ^80^ ^]^ Upon solar illumination, photothermal heating contributes to the thermal excitation of charge carriers, effectively reducing recombination and accelerating surface reactions. Electrons transferred to the conduction bands react with O_2_ to form •O_2_^−^, with H_2_O_2_ converted to •OH via photolysis. Both radical species are highly oxidative and attack the organic pollutants. Thus, the hybrid's broad‐spectrum absorption, efficient charge transfer, and heat‐induced mass transport all cooperate to produce abundant ROS and rapidly mineralize dyes and pesticides.

#### 2.6.2 Plant Growth Assessment

To further assess the environmental safety of the treated effluent, a 7‐day growth assay using mung bean (Vigna radiata) plants was performed (Figure 7l). The results clearly demonstrate the phytotoxicity of the untreated dye solutions. When irrigated with 10 mg/l dye solutions, the seedlings exhibited severely suppressed growth. Stem heights were limited to 8.4 cm for MO, 8.9 cm for MB, and only 4.8 cm for CV, accompanied by weak germination vigor, pale chlorotic leaves, and poor root development. These trends are fully consistent with prior studies reporting that synthetic dyes impede nutrient uptake, inhibit photosynthesis, and impair metabolic activity in plants.^[^ ^81^, ^82^ ^]^ In sharp contrast, plants watered with Bi_2_Te_3_@CdS‐treated effluent showed a dramatic recovery in growth performance, reaching stem heights comparable to the control group irrigated with DI water (≈12.7 cm). Specifically, seedlings irrigated with the treated MO, MB, and CV solutions achieved average heights of 12.5, 12.1, and 12.3 cm, respectively, representing nearly complete restoration (≈95–98%) of normal growth relative to the control. These values reflect that hybrid‐coated PU foam successfully removes toxic dye molecules, thereby eliminating the major stress factors responsible for growth inhibition.

The radial plot in Figure 7m further visualizes this striking contrast, while untreated solutions cluster near the lower growth axis (4–9 cm), the treated samples shift toward the outer ring (12–14 cm), nearly overlapping with the control in all evaluated parameters. This quantitative improvement underscores that the catalyst‐treated water not only meets pollutant‐removal criteria but is also biologically compatible for plant irrigation. Collectively, these biological assays provide compelling evidence that Bi_2_Te_3_@CdS‐coated PU foam produces an effluent that is environmentally benign and supportive of healthy plant development. When combined with its demonstrated broad‐spectrum light absorption, strong photothermal conversion, efficient charge separation, and high pollutant degradation capability, the hybrid foam represents a robust and sustainable strategy for solar‐driven wastewater remediation. Importantly, the system addresses both contaminant elimination and ecological safety, reinforcing its potential for practical deployment in environmentally sensitive applications.

### 2.7 Antibacterial Window

**Figure 8:**
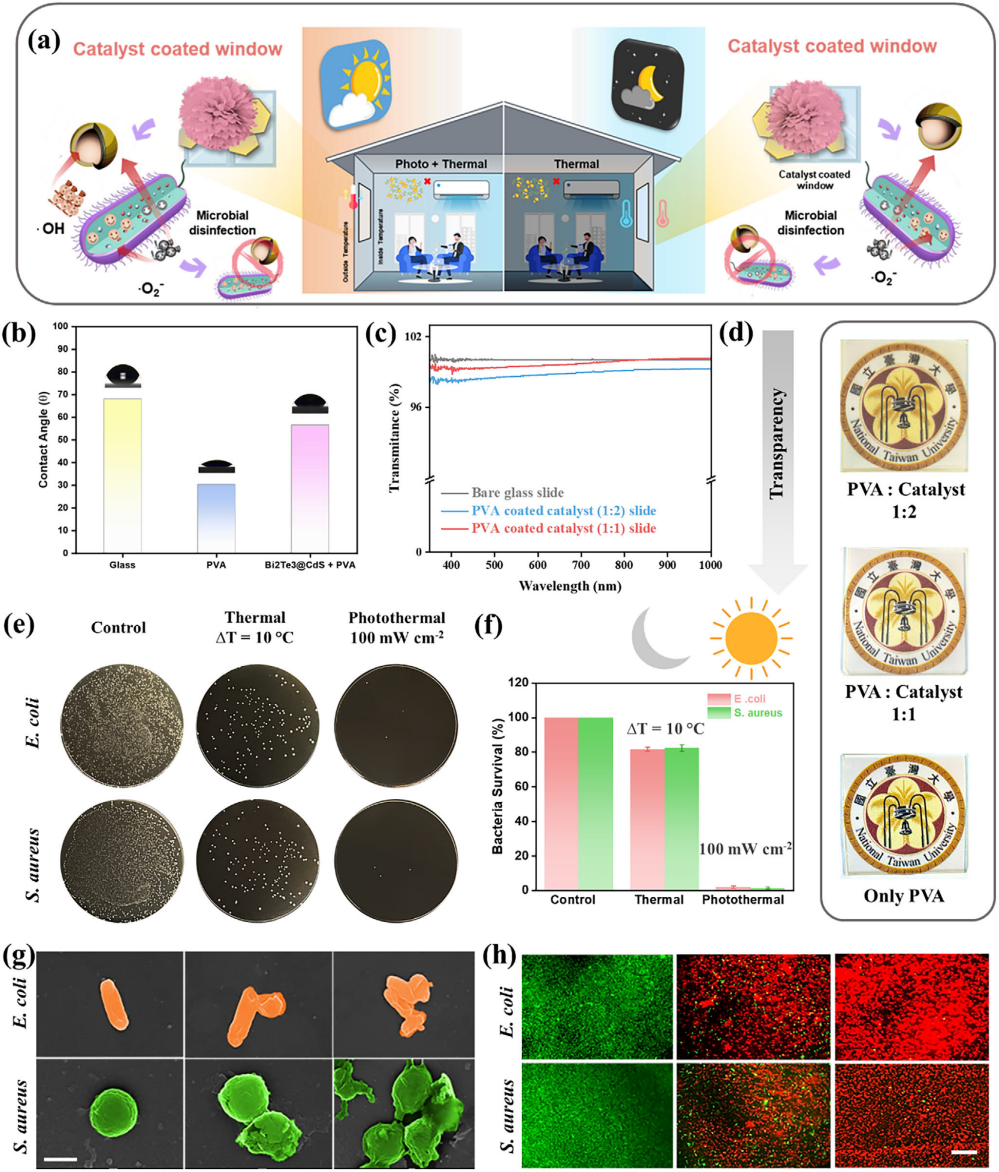
a) Schematic of Bi_2_Te_3_@CdS catalyst‐coated glass enabling continuous antimicrobial disinfection via solar and thermal activation. b) Contact angle measurements with PVA and catalyst coatings. c) Optical transmittance spectra of catalyst‐coated glass. d) Photographs demonstrating transparency of glass slides. e) Antibacterial plate assay showing *E. coli* and *S. aureus* colonies after treatment. f) Bacterial survival under different conditions. g) SEM images of bacteria after exposure to catalyst‐coated glass. Scale bar: 1 µm. h) Live/dead fluorescence staining confirming bacterial killing by catalyst‐coated glass. Scale bar: 100 µm. Results are plotted as means ± SD (*n* = 3).

**Figure** **8**a illustrates the application of Bi_2_Te_3_@CdS‐coated glass as a window, which can harness both solar (photo) and thermal energy to generate ROS such as •O_2_^−^ and •OH. These ROS are responsible for microbial disinfection, effectively inactivating bacteria on the glass surface. The schematic illustrates two operating modes: daytime (photothermal) and nighttime (thermal), both of which contribute to continuous antimicrobial protection within the room. Figure 8b depicts the water contact angle measurements for bare glass, PVA‐coated glass, and Bi_2_Te_3_@CdS + PVA‐coated glass. The contact angle is an indicator of surface wettability. Bare glass exhibits a high contact angle (hydrophobic), whereas the PVA coating significantly reduces the angle (making it hydrophilic). Bi_2_Te_3_@CdS + PVA coating maintains moderate wettability, which is beneficial for uniform catalyst dispersion and interaction with aqueous bacterial suspensions.

For the transmittance, Figure 8c shows a graph comparing optical transmittance spectra of bare glass and Bi_2_Te_3_@CdS/PVA‐coated glass (at 1:1 and 1:2 ratios). All samples exhibit high transmittance (>95%) across the visible to near‐infrared region (350‐1000 nm), indicating that the catalyst coating does not significantly compromise the transparency of the glass, making it suitable for window applications. Figure 8d provides photographic evidence of the transparency of coated glass slides, showing clear visibility through PVA: Bi_2_Te_3_@CdS‐coated glass at both 1:2 and 1:1 ratios, as well as PVA‐only control. This further confirms that the catalyst layer maintains high optical clarity.

The antibacterial efficacy against *E. coli* and *S. aureus* was demonstrated by the results (glass coated with 1:1 ratio of PVA to catalyst) shown in Figure 8e. Three conditions are compared for a 15 min treatment: control (no treatment), thermal treatment (ΔT = 10 °C), and combined photothermal treatment (100 mW/cm^2^). The control plates show dense bacterial colonies, while thermal treatment reduces colony numbers (≈80%). The photothermal treatment nearly eliminates bacterial growth (≈99%), demonstrating the strong antibacterial efficacy of the catalyst‐coated glass under simulated sunlight. Figure 8f presents a bar graph quantifying bacterial CFU for *E. coli* and *S. aureus* under the same three conditions as shown in Figure 8e. To simulate night‐time conditions in the absence of sunlight, antibacterial efficacy was evaluated at controlled temperature gradients of ΔT = 5 °C and 15 °C, as shown in Figure S16 (Supporting Information). These conditions resulted in bacterial survival rates ranging from ≈70% to over 99% cell death for ΔT = 5 °C and 15 °C, respectively. These results quantitatively confirm the catalyst's robust efficacy across varying thermal gradients, highlighting its potential for continuous environmental disinfection regardless of solar irradiation.

SEM images of *E. coli* and *S. aureus* after exposure to the catalyst‐coated glass are shown in Figure 8g. The untreated bacteria (left) appear intact, while those exposed to the catalyst (right) exhibit significant morphological damage, including cell wall disruption and deformation, indicating effective bacterial inactivation by the generated ROS. Figure 8h shows fluorescence microscopy images of both bacteria stained with live/dead viability dyes after treatment. Live bacteria fluoresce green, while dead bacteria fluoresce red. The control samples exhibit predominantly green fluorescence, indicating the presence of viable cells. In contrast, samples treated with the catalyst‐coated glass under photothermal conditions display mostly red fluorescence, confirming extensive bacterial death.

## 3 Conclusion

We have demonstrated a multifunctional Bi_2_Te_3_@CdS hybrid that harnesses dual photothermal and thermocatalytic effects for solar‐driven remediation and disinfection. Heterojunction between narrow‐gap Bi_2_Te_3_ and visible‐light CdS yields broad solar absorption and highly efficient charge separation, as evidenced by dramatic PL quenching and prolonged carrier lifetimes. Under one‐sun illumination, the material heats locally (≈30–50 °C), accelerating wastewater treatment kinetics and enabling continuous generation of ROS. The result is rapid pollutant mineralization (e.g., ≈97% dye removal) along with potent antibacterial activity. Solar illumination alone achieves ≈99% antibacterial efficiency for *E. coli* and *S. aureus* in 15 min, and even low‐level light produces sterilizing heat. Importantly, floating PU foam ensures easy catalyst recovery and long‐term stability, with ≈90% activity retained after 5 reuse cycles. These findings illustrate key scientific insights: rational band‐engineering and interface design can fully exploit the solar spectrum, and photothermal heating can be used not just for water evaporation but also to augment photocatalytic reactions and to disrupt microbial membranes. Bi_2_Te_3_@CdS hybrid platform thus exemplifies a truly self‐sufficient remediation system. The present hybrid foam and coating could be applied in modular water treatment units or as clear antibacterial window films. The multifunctionality of wastewater detoxification, airborne or surface pathogen control, energy efficiency, and robustness (durability, recyclability) position this system as a promising strategy for sustainable environmental and public‐health technologies.

## Conflict of Interest

The authors declare no conflict of interest.

## Supporting information



Supporting Information

## Data Availability

The data that support the findings of this study are available from the corresponding author upon reasonable request.
