# Supplementary material for: Hot Electron‐Assisted Noble‐Metal‐Free Synergistic Photothermal Catalyst for Solar‐Driven Wastewater Remediation and Microbial Disinfection
Source: Adv Sci (Weinh). 2025 Dec 12;13(11):e15018. doi: 10.1002/advs.202515018 (PMC12931260; doi:10.1002/advs.202515018)
Supplement: Supplementary file 1 — Supporting Information [file ADVS-13-e15018-s001.docx]

**Hot Electron-Assisted Noble-Metal-Free Synergistic Photothermal Catalyst for Solar-Driven Wastewater Remediation and Microbial Disinfection**

Manish Kumar Sharma^1,2,7^, Bishal Kumar Nahak^1^, Parag Parashar^1^, Uday Kumar Singh^1^, Arshad Khan^3^, Jaba Roy Chowdhury^1^, Parthasarathi Pal^1^, Dongwhi Choi^6^, Hae Gyun Lim^7^, Yu-Lun Chueh^2,4,5,^*, and Zong-Hong Lin^1,6^^,^*

^1^ Department of Biomedical Engineering, National Taiwan University, Taipei 10617, Taiwan

^2^Department of Materials Science and Engineering, National Tsing Hua University, Hsinchu 30013, Taiwan

^3^International Intercollegiate PhD Program, National Tsing Hua University, Hsinchu 30013, Taiwan

^4^Department of Physics, National Sun Yat-Sen University, Kaohsiung, 80424, Taiwan

^5^Department of Materials Science and Engineering, Korea University, Seoul 02841, Republic of Korea

^6^Department of Mechanical Engineering (Integrated Engineering Program), Kyung Hee University, Gyeonggi-do 17104, Republic of Korea

^7^Department of Biomedical Engineering and Smart Gym‑Based Translational Research Center for Active Senior Healthcare, Pukyong National University, Busan 48513, Republic of Korea

*Corresponding author:

Email: ylchueh@mx.nthu.edu.tw (Yu-Lun Chueh)

Email: zhlin@ntu.edu.tw (Zong-Hong Lin)

**Supporting Information**

**Experimental Section**

Synthesis of Bi_2_Te_3_ and CdS nanoparticles

Bi_2_Te_3_ and CdS were synthesized separately via hydrothermal reactions using their respective salt precursors (190°C, 3 h and 200°C, 6 h). For Bi_2_Te_3_ NPs, bismuth nitrate pentahydrate ((Bi(NO_3_)_3_·5H_2_O), 0.194 g) and sodium tellurite (Na_2_TeO_3_, 0.133 g) served as sources, while cadmium acetate (Cd(CH_3_COO)_2_, 1.66 g) and thiourea ((NH_2_)_2_CS, 0.49 g) were used to form CdS microflowers. Post-synthesis, both nanoparticles were purified and dried.

Synthesis of Bi_2_Te_3_@CdS hybrid

Bi_2_Te_3_@CdS hybrid was synthesized by dispersing Bi_2_Te_3_ and CdS powders in ethanol at weight ratios of 1:1, 1:2, and 1:4 (Bi_2_Te_3_:CdS, w/w). The mixture was stirred continuously at 500 rpm and maintained at 60°C overnight to promote effective heterojunction formation through physical adsorption and interfacial interactions. After stirring, the hybrid material was collected by centrifugation or filtration, washed several times with ethanol to remove unbound particles, and then dried under vacuum or in an oven at 60°C. This straightforward method yielded a well-integrated Bi_2_Te_3_@CdS composite with tunable composition, suitable for further characterization and catalytic applications.

PU foam coated with Bi_2_Te_3_@CdS hybrid

PU foam substrates were first cut into discs measuring 3 cm in diameter and 5.5 mm in thickness, followed by a thorough cleaning process to remove surface contaminants. To enhance the adhesion of Bi_2_Te_3_@CdS hybrid catalyst, cleaned PU foams were subjected to low-pressure oxygen plasma treatment for varying durations, which increased surface hydrophilicity and introduced reactive functional groups. Subsequently, 5 mg of Bi_2_Te_3_@CdS hybrid was dispersed in ethanol and uniformly deposited onto plasma-treated PU foam surfaces. The coated foams were then dried overnight at 60°C to ensure stable immobilization of the hybrid material, resulting in a uniformly anchored Bi_2_Te_3_@CdS coating on PU foam (Figure S12).

Glass coated with Bi_2_Te_3_@CdS hybrid

Glass substrates coated with Bi_2_Te_3_@CdS hybrid were prepared using a polymer-assisted spin coating method. Initially, 0.2 mg of sodium alginate was dissolved in 3 ml of deionized (DI) water and stirred until fully mixed. Separately, a 5 wt% polyvinyl alcohol (PVA) solution was prepared by dissolving 0.5 g of PVA in 10 ml of DI water at 80°C under continuous stirring for 30 min, followed by cooling to room temperature. The sodium alginate solution was then gradually added to PVA solution under constant stirring to form a homogeneous polymer blend. Subsequently, 0.2 ml of glutaraldehyde was added dropwise to the polymer mixture while stirring to induce crosslinking. Different ratios of this polymer solution and Bi_2_Te_3_@CdS hybrid (1:1 and 1:2, w/w) were prepared and deposited onto pre-cleaned glass slides by spin coating. Prior to coating, the glass substrates were thoroughly cleaned by immersing them in acetone and then rinse with ethanol and then dry in a vacuum chamber. Coated slides were then dried to obtain uniform and stable Bi_2_Te_3_@CdS hybrid films embedded within polymer matrix.

H_2_O_2_ Accumulation and Quantification

H_2_O_2_ quantification was performed using the Amplex Red assay. In this method, a catalyst-coated foam was immersed in DI water to carry out catalytic cycles for H_2_O_2_ generation. Subsequently, 270 µl of catalytic solution was mixed with 30 µl of Amplex Red reagent, followed by the addition of 3 µl horseradish peroxidase (HRP) solution. The mixture was incubated for 30 min to allow the reaction to proceed, after which fluorescence measurements were taken at various time intervals. For calibration, a 30% aqueous H_2_O_2_ stock solution with a molarity of 9.8 M was serially diluted to prepare standard solutions of 0 mM, 0.625 mM, 1.25 mM, 2.5 mM, 5 mM, 10 mM, 20 mM, and 30 mM (Figure S16). These calibration standards were used to generate a standard curve for accurate quantification of H_2_O_2_ concentrations in the experimental samples.

EPR analysis for free radicals

EPR spectroscopy was employed to analyze the generation of •OH and •O_2_⁻. To trap these radicals, DMPO/water and DMPO/methanol solutions were used as spin-trapping agents, respectively. For each measurement, 1 mg of catalyst was dispersed in 2 ml of a 100 mM aqueous DMPO solution (with an additional 1 mM methanol for •O_2_⁻ detection). The suspensions were exposed to simulated solar irradiation for 15 min to induce radical formation. After 15 min of treatment, catalytic solution was filtered and analyzed using Bruker EPR-plus spectrometer (Germany) operating in X-band at 9.842442 GHz. The instrumental parameters included an amplitude modulation of 1.25 G, microwave power of 31.88 mW, modulation frequency of 100 kHz, conversion time of 62.5 ms, and a time constant of 0.01 ms.

Dye degradation experiment

Aqueous solutions of MB, MO, and CV dyes were prepared at a concentration of 10 mg/l, with a volume of 50 ml for each solution. Catalyst coated PU foam was used for each dye solution and subjected to solar simulation to facilitate efficient interaction between the catalyst surface and dye molecules. Samples were collected at time intervals ranging from 0 min to 60 min to monitor degradation process. The residual dye concentrations at each interval were quantitatively analyzed using UV-visible spectroscopy (JASCO V-670), by measuring the characteristic absorption peaks of the respective dyes.

CPS degradation experiment

CPS stock solution was prepared by dissolving 100 mg of CPS in 100 ml of acetonitrile, which was subsequently diluted with water to obtain a final concentration of 10 mg/l in 50 ml aqueous solution. Catalyst-coated PU foam was immersed in CPS solution and exposed to simulated solar irradiation to promote catalytic degradation. Samples were collected at 10 min intervals over a total duration of 40 min to monitor the degradation. The residual CPS concentration at each time point was determined using above mentioned UV-Vis spectroscopy.

Preparation of bacterial solutions

*E. coli* K-12 DH5α and *S. aureus* ATCC 29213 bacterial cells were cultured in LB medium at 37°C for 16 hours in an incubator with continuous shaking at 180 rpm. After incubation, the cultures were centrifuged at 5000 rpm for 10 min to remove LB medium from the supernatant. The bacterial pellets were then diluted in PBS to achieve optical densities of 0.06 and 0.3 at 670 nm. Next the bacterial solutions were diluted with PBS to prepare a concentration of 2 × 10^6^ CFU/ml for antibacterial studies.

Antibacterial experiment

To determine the antibacterial activity, bacterial samples were treated in three different groups: control, thermal, and photothermal. After treatment, bacteria solutions (aliquots of 100 µl) were collected and spread on solid LB-agar plates and incubated overnight at 37°C for colony growth. After incubation, the photographs of the plates were taken, and bacterial colonies were counted using ImageJ software for quantitative analysis of antibacterial activity. Further, the bacterial survival rates were calculated using the following equation.

$$Bacteria survival \left( \% \right)=\frac{C_{0}-C}{C_{0}}\times100\%$$

where, C_0_ is the initial concentration of bacteria and C is the remaining concentration of bacteria after each treatment.

For SEM analysis, the treated bacterial samples were first fixed in a 4% glutaraldehyde solution for 30 min, followed by three washes with 0.01 M PBS. The samples were then dehydrated through a graded ethanol series (25%, 35%, 45%, 55%, 65%, 75%, 85%, and 95% v/v), with each step lasting 10 min. After dehydration, 10 μl aliquots of the samples were placed onto slides, vacuum-dried overnight, and subsequently analyzed using SEM.


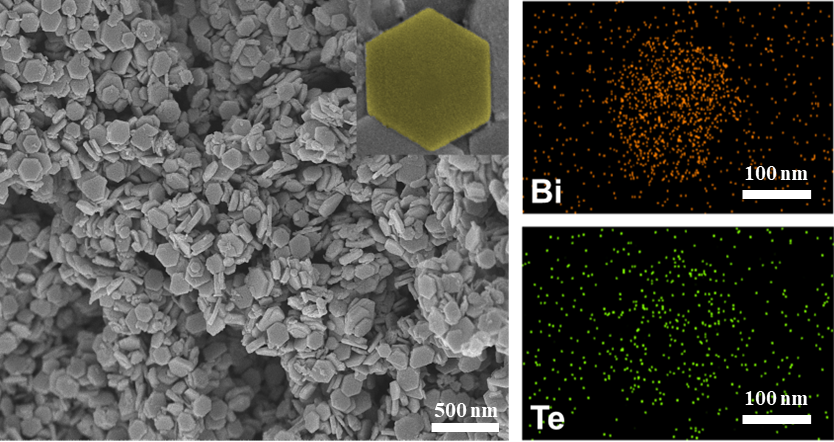


**Figure S1.** SEM and EDX of Bi_2_Te_3_ NPs.


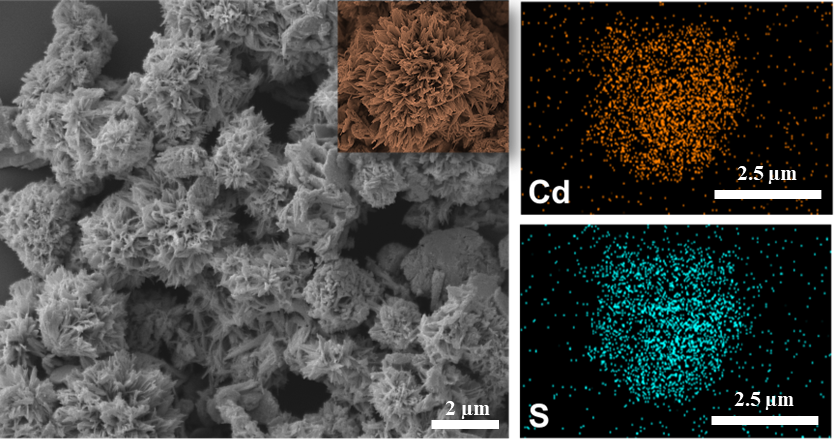


**Figure S2.** SEM and EDX of CdS microflowers.


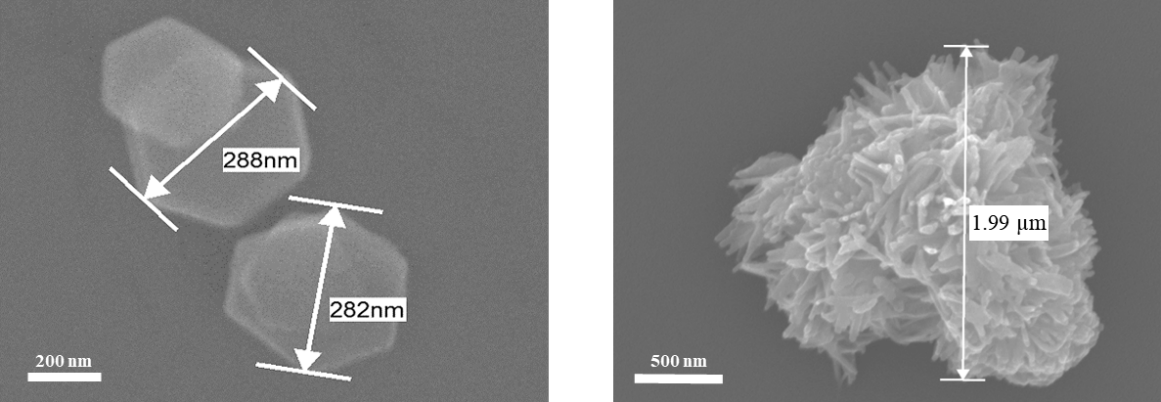

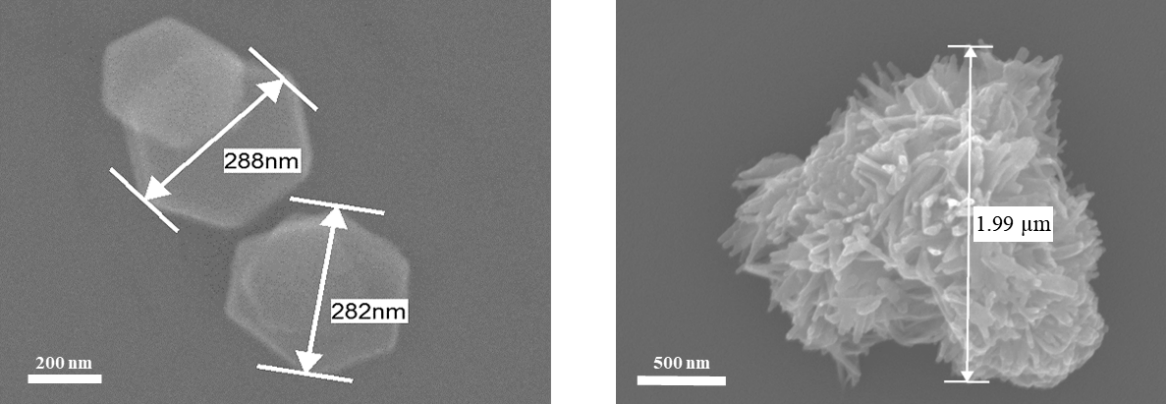


**Figure S3.** SEM for particle size measuerement of distribution of Bi_2_Te_3_ and CdS nanoparticles.


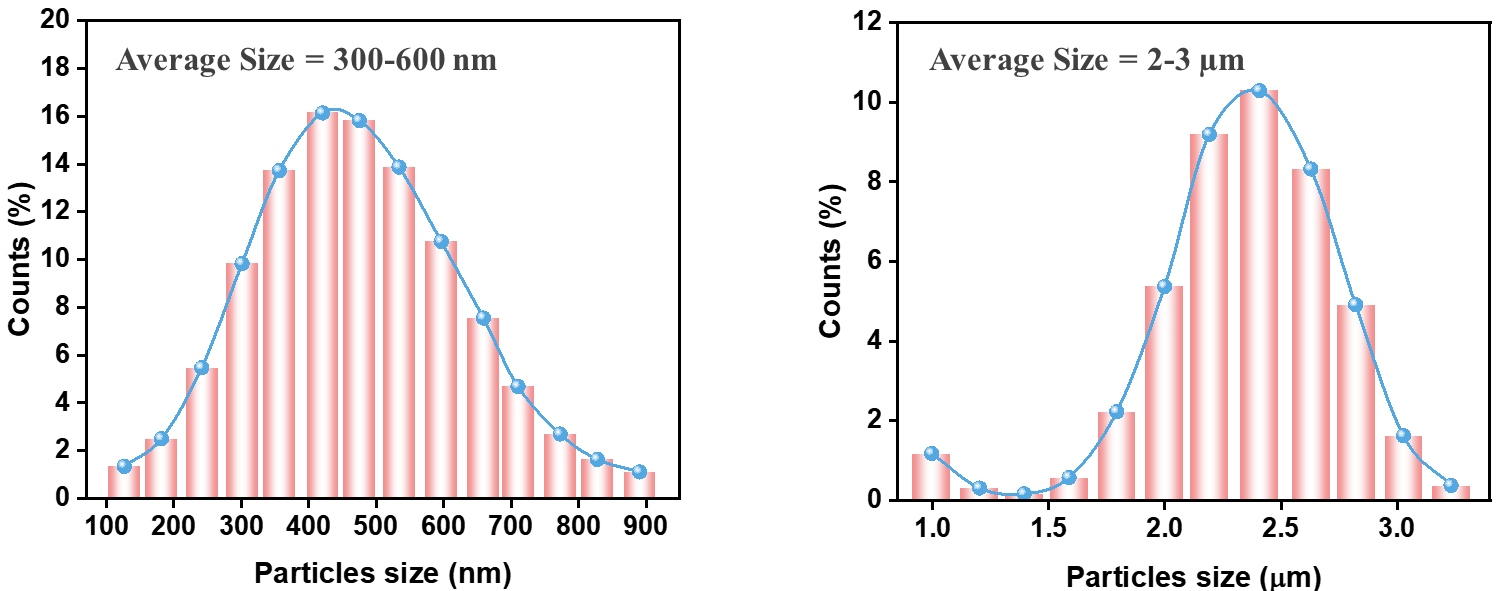


**Figure S4.** Particle size distribution of Bi_2_Te_3_ and CdS nanoparticles.


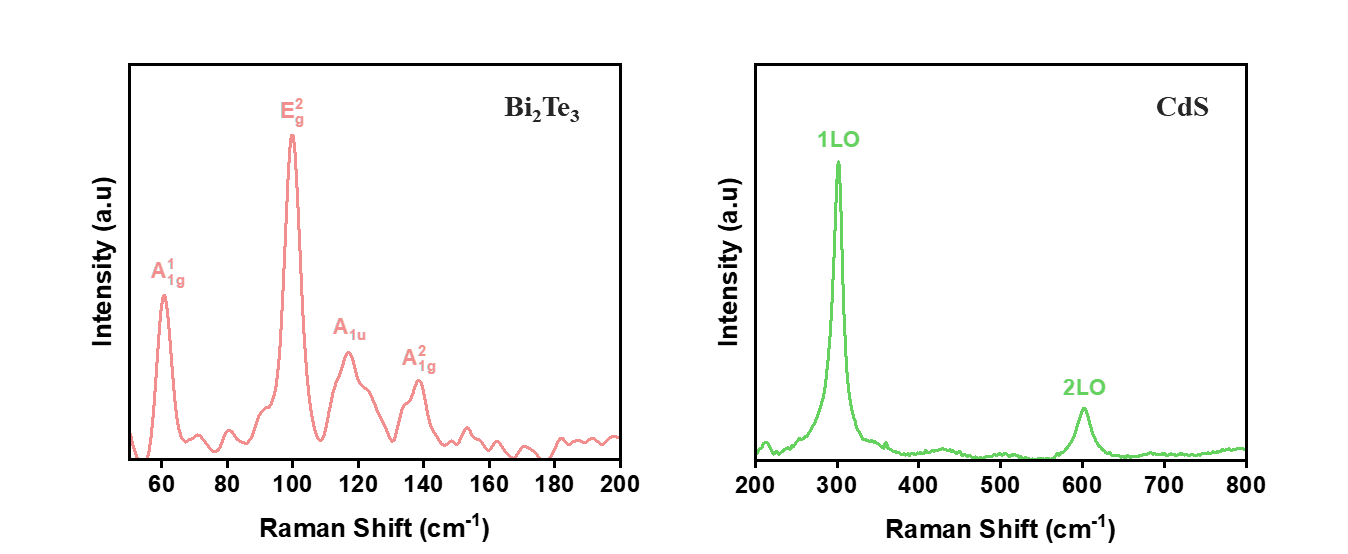


**Figure S5**. Raman Spectra of Bi_2_Te_3_ and CdS nanoparticles.


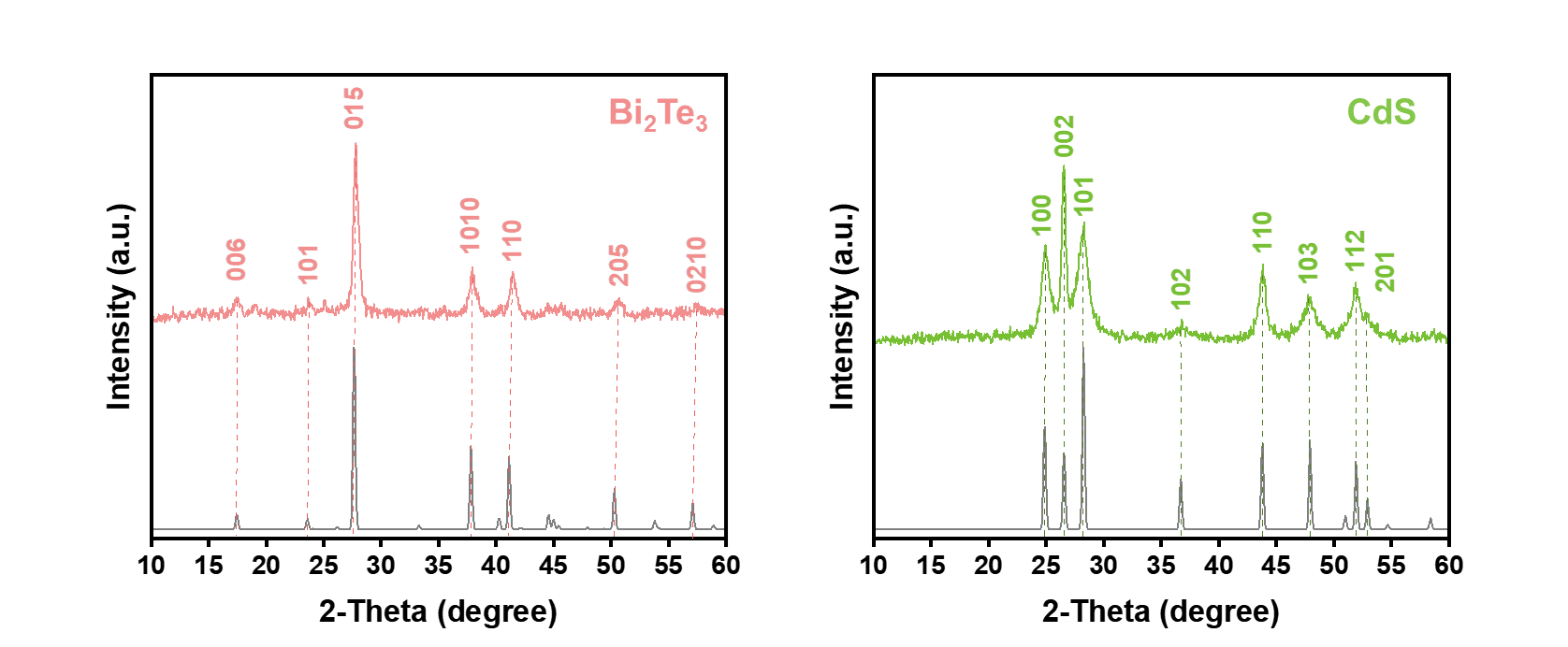


**Figure S6.** XRD patterns of the as-synthesized Bi_2_Te_3_ and CdS nanoparticles and corresponding standard JCPDS card (15-0863) and (892944) respectively.


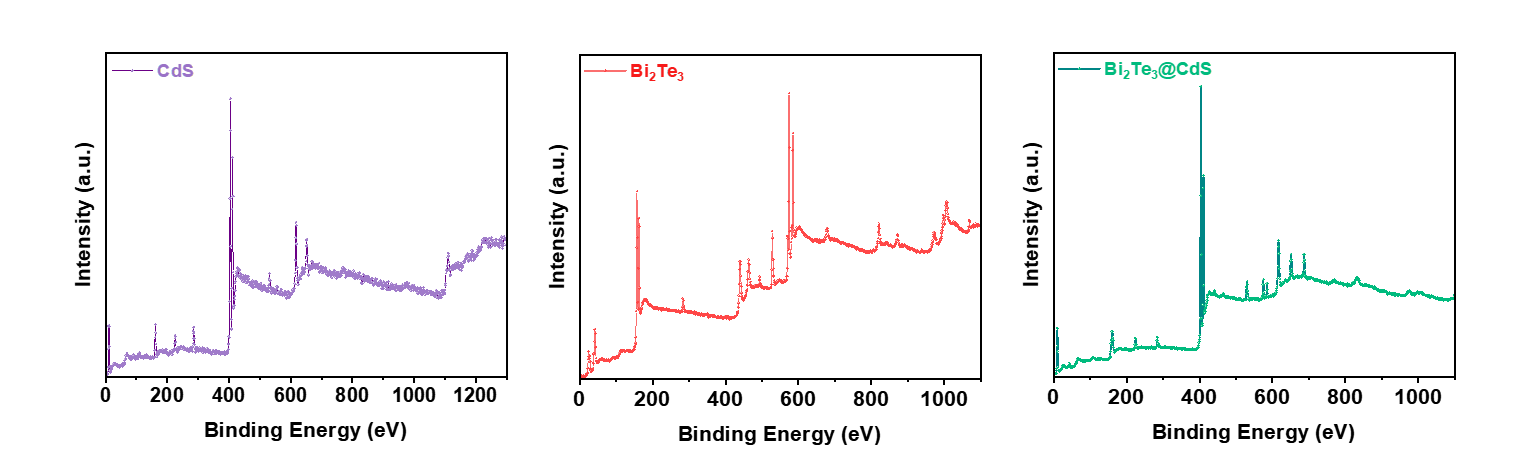


**Figure S7.** XPS survey spectrum of CdS, Bi_2_Te_3_ and Bi_2_Te_3_@CdS hybrid nanoparticles.


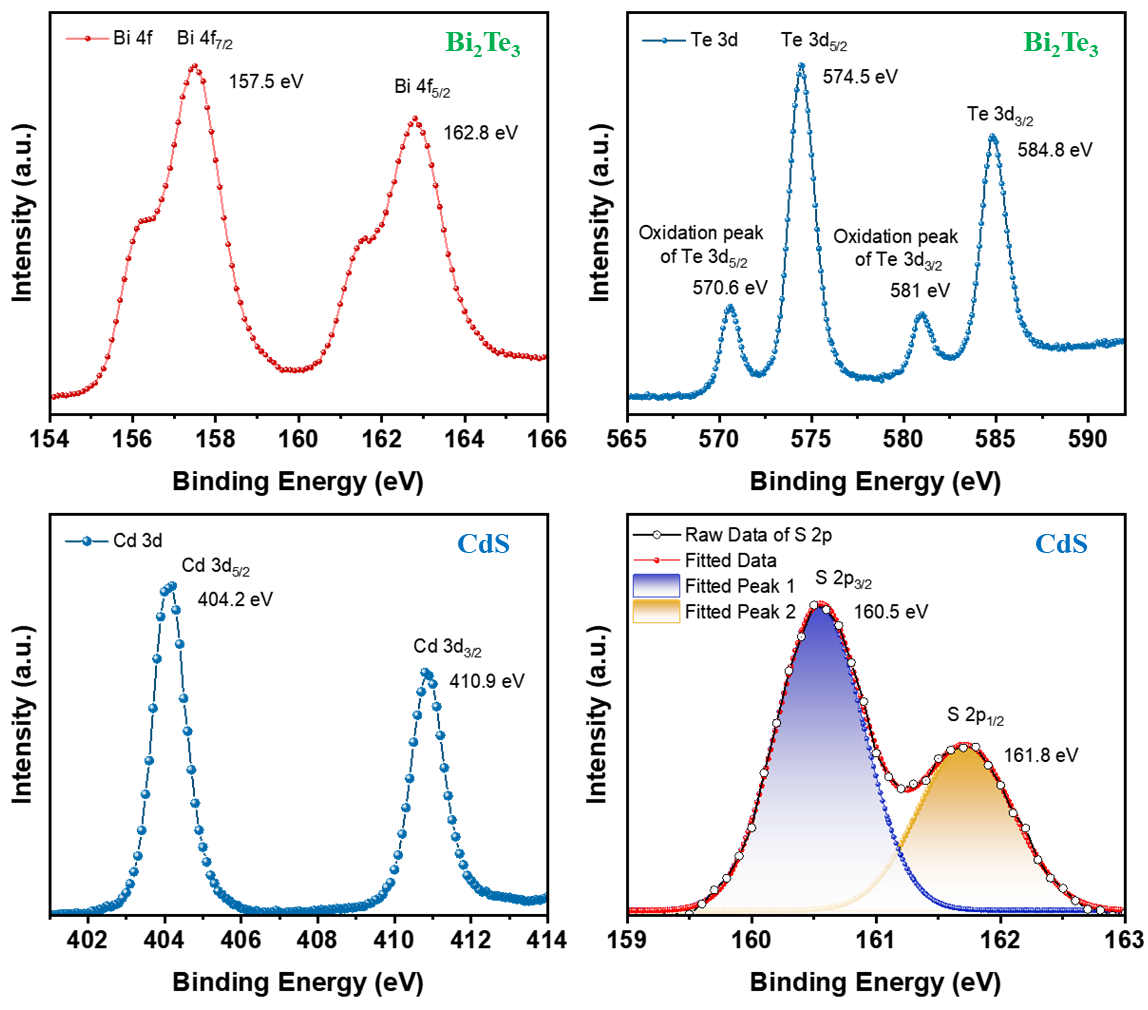


**Figure S8.** High-resolution XPS spectra for Bi 4f and Te 3d of Bi_2_Te_3_ and Cd 3d and S 2p of CdS nanoparticles.


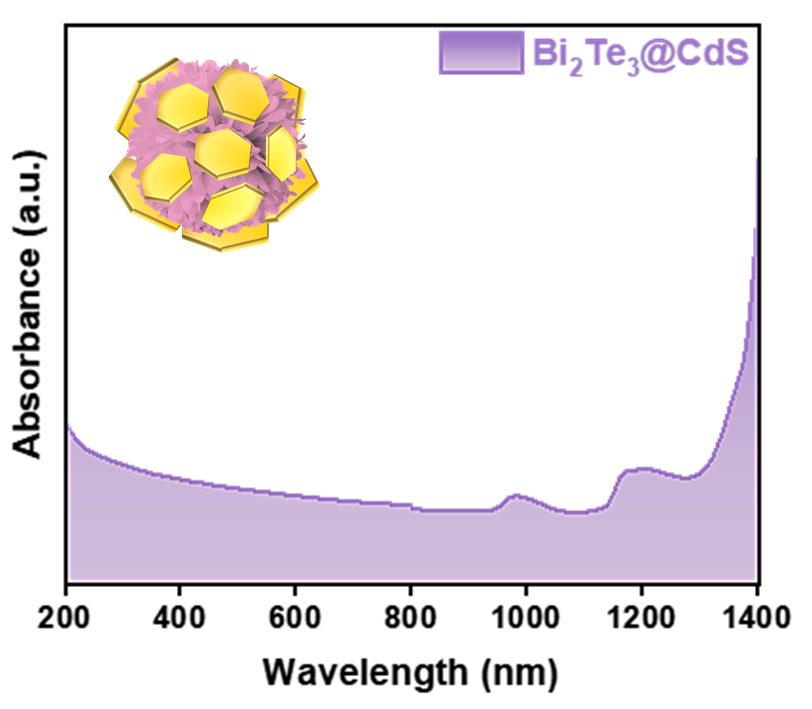


**Figure S9.** UV-vis absorbance of Bi_2_Te_3_@CdS hybrid nanoparticles.


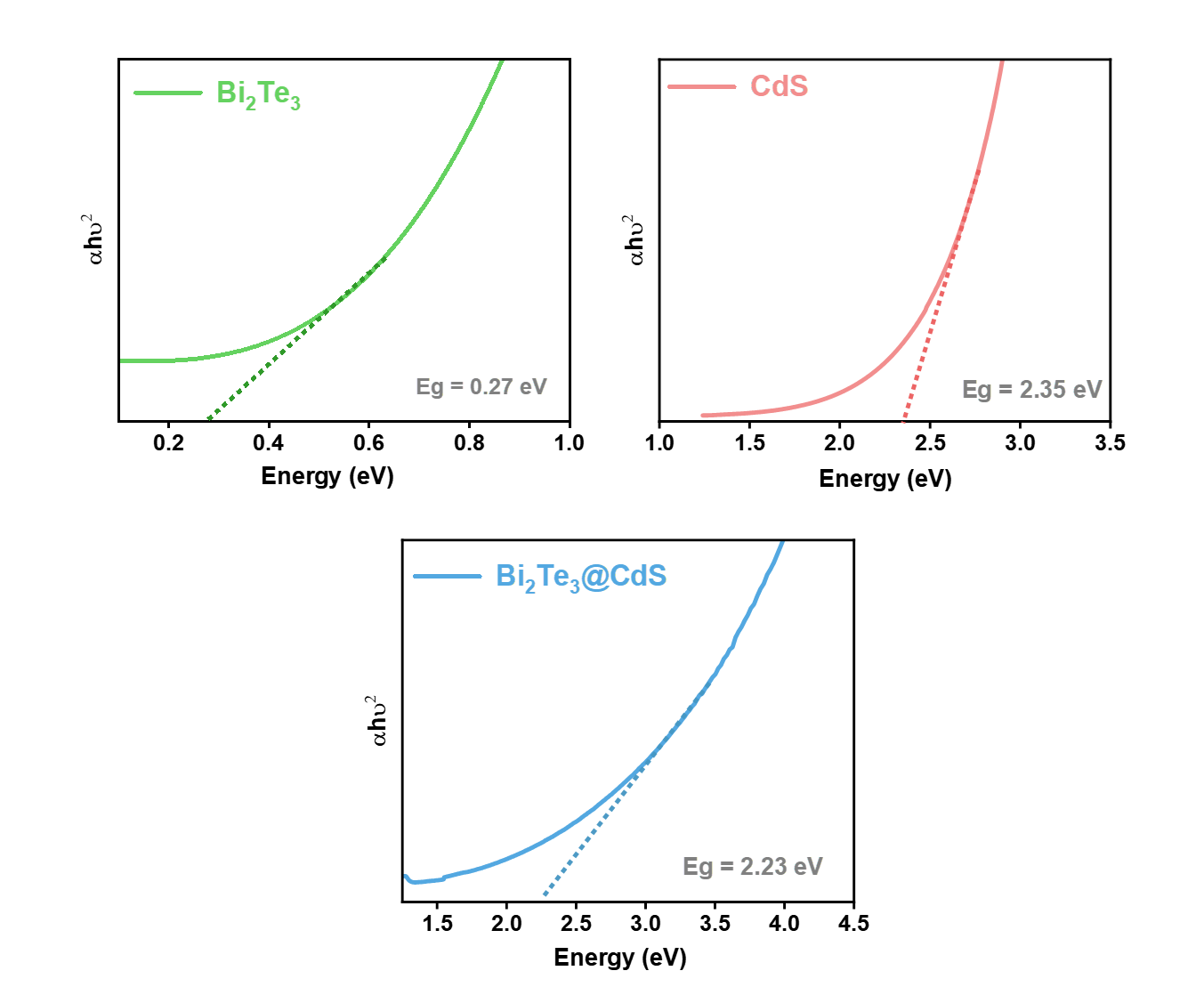


**Figure S10.** Band gap calculation of Bi_2_Te_3_, CdS, and Bi_2_Te_3_@CdS hybrid nanoparticles.

**Table S1.** Average lifetime intensity of Bi_2_Te_3_, CdS, and Bi_2_Te_3_@CdS hybrid nanoparticles.

| **Samples** | **Amplitude**  **(in counts)** | **Lifetime**  **(in ns)** | **τ_Av_**  **(Intensity in ns)** |
| --- | --- | --- | --- |
| Bi_2_Te_3_ | A_1_ = 251.7  A_2_= 2543.3  A_3_ = 8006 | τ_1_ **=** 3.717  τ_2_ **=** 1.1496  τ_3_ **=** 0.23936 | 1.263 |
| CdS | A_1_ = 74.17  A_2_= 941.7  A_3_ = 5988 | τ_1_ **=** 11.548  τ_2_ **=** 1.1583  τ_3_ **=** 0.11832 | 4.231 |
| Bi_2_Te_3_@CdS | A_1_ = 145.74  A_2_= 1275  A_3_ = 3596 | τ_1_ **=** 14.955  τ_2_ **=** 1.4206  τ_3_ **=** 0.1228 | 7.947 |


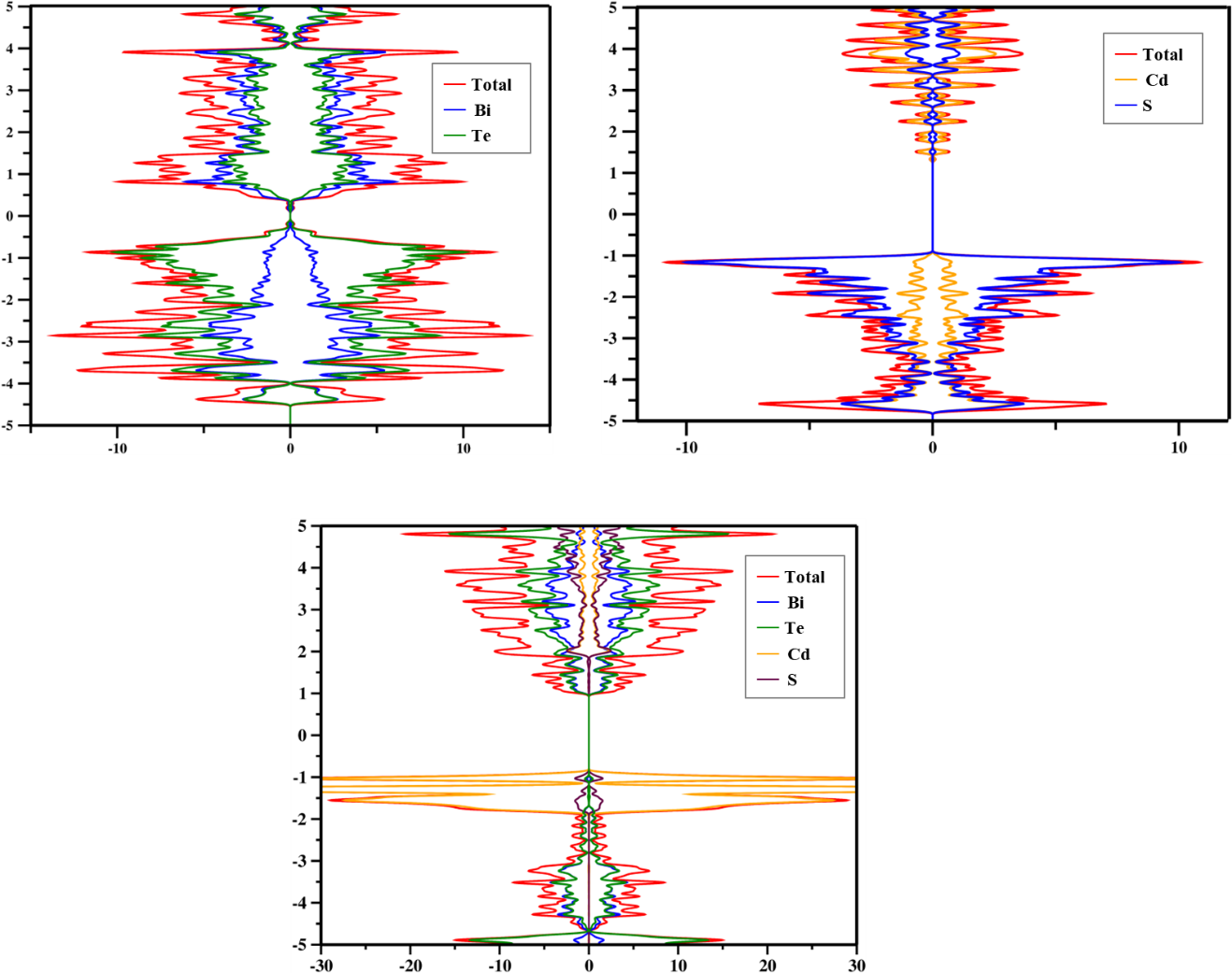


**Figure S11.** Total DOS of Bi_2_Te_3_, CdS, and Bi_2_Te_3_@CdS hybrid.


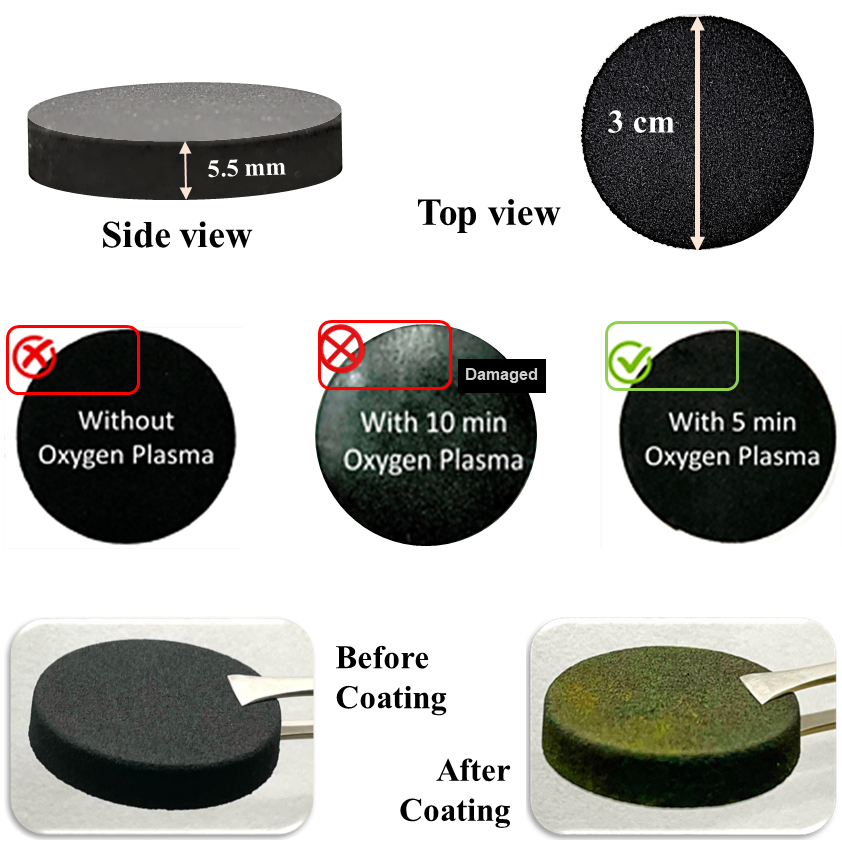


**Figure S12.** Optical photo of PU foam with plasma treatment at different duration and coating with Bi_2_Te_3_@CdS hybrid.


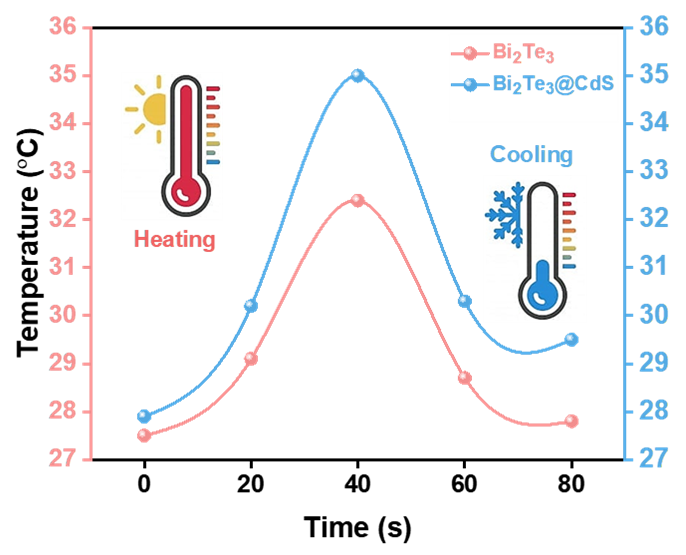


**Figure S13.** Temperature comparison of Bi_2_Te_3_ and Bi_2_Te_3_@CdS hybrid for single thermal cycle.


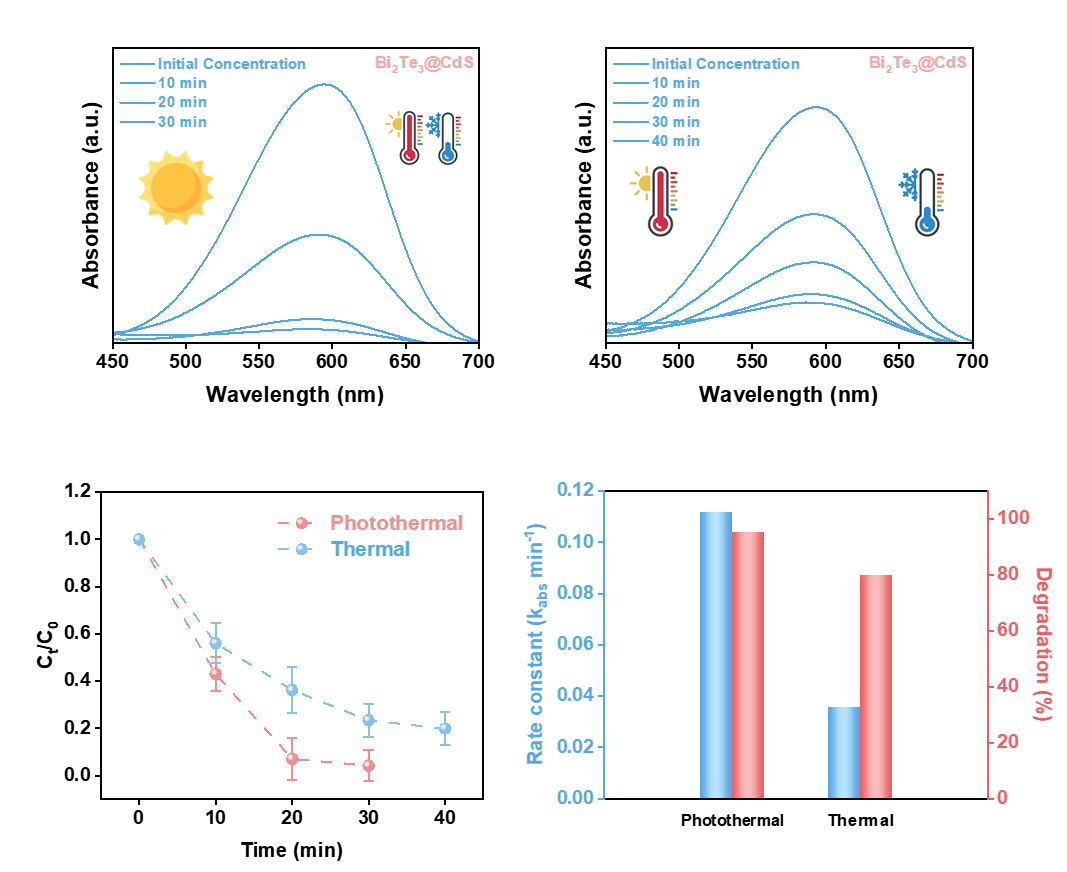


**Figure S14**. Comparison of photothermal and thermal degradation of MB dye with rate constant and degradation efficiency.


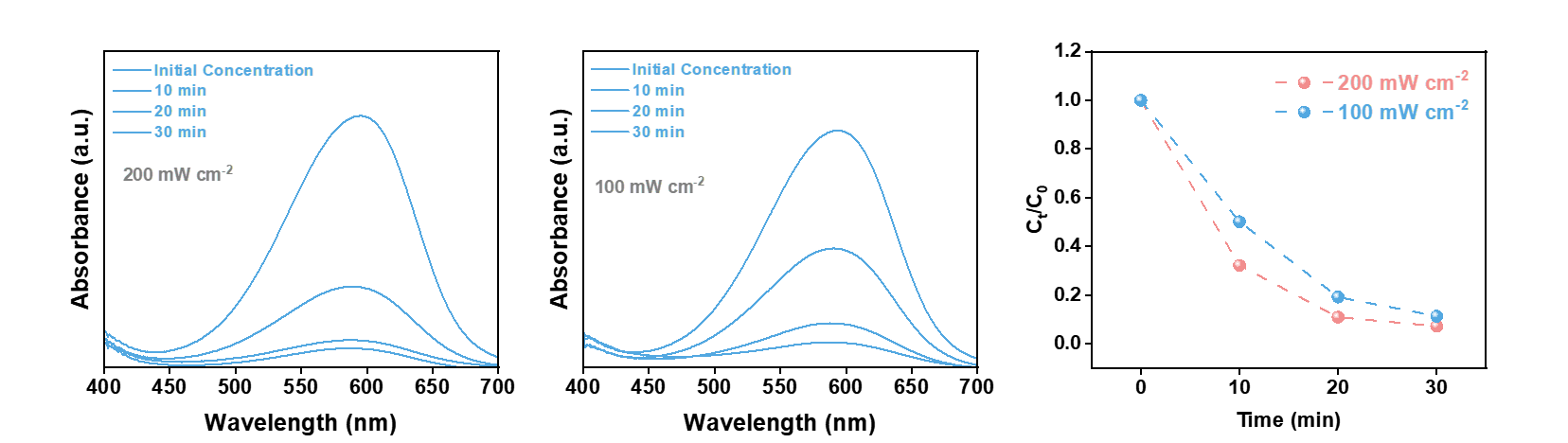


**Figure S15.** Comparison of different solar intensity for degradation of MB dye with efficiency.

**
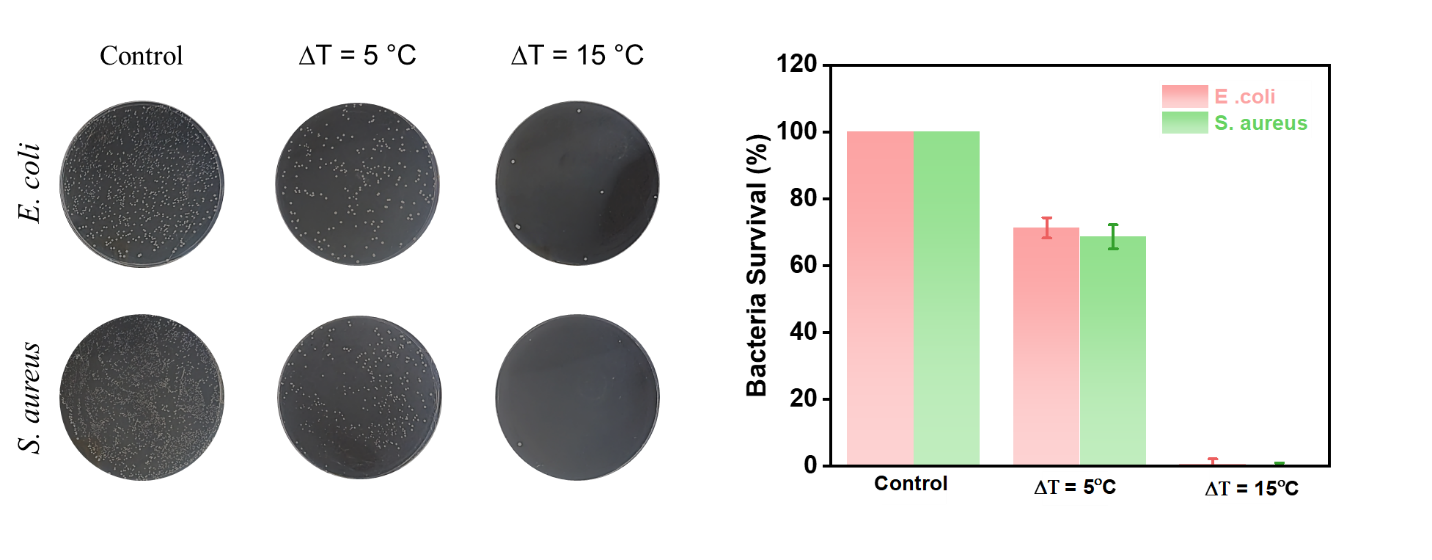
**

**Figure S16.** Disinfection performance of Bi_2_Te_3_@CdS hybrid under different applied temperature differences. Results are plotted as means ± SD (n = 3).

**
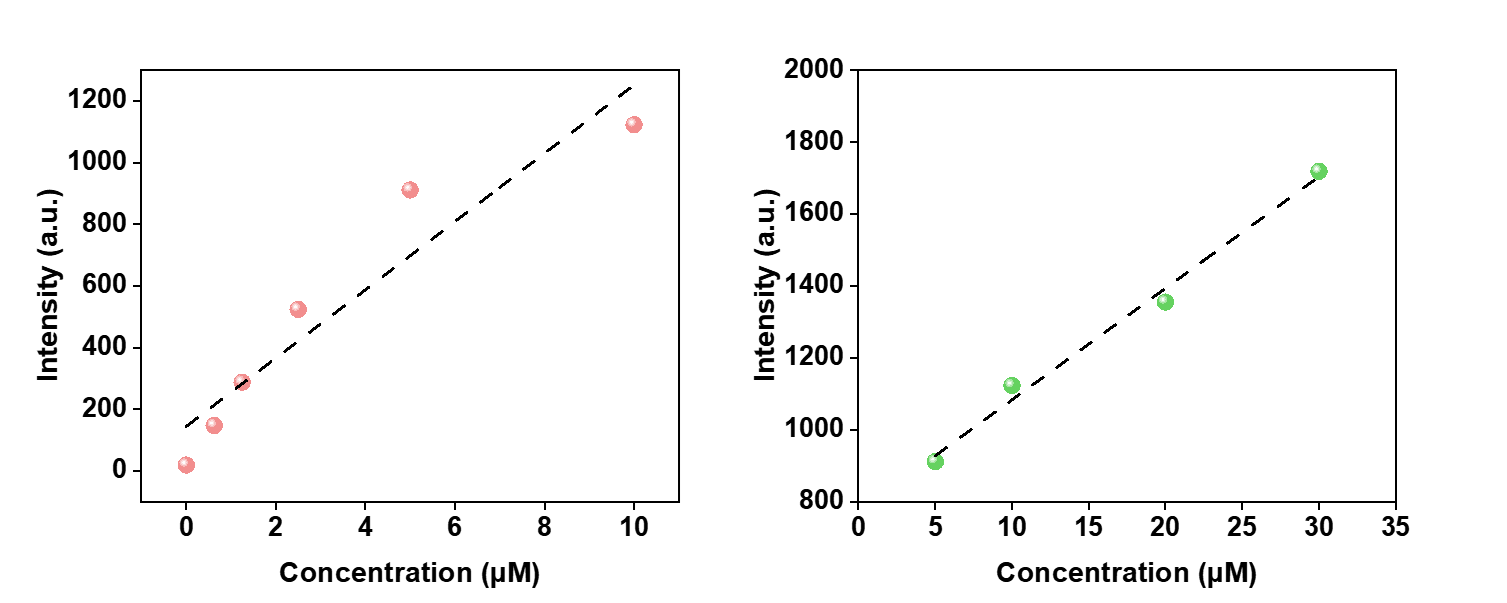
**

**Figure S17.** H_2_O_2_ quantification from stock solution by Amplex Red method.
